# A simple intuitive method for seeking intersections of hyperbolas for acoustic positioning biotelemetry

**DOI:** 10.1371/journal.pone.0276289

**Published:** 2022-11-09

**Authors:** Junichi Takagi, Hirotaka Kanazawa, Kotaro Ichikawa, Hiromichi Mitamura

**Affiliations:** 1 National Institute of Polar Research, Tokyo, Japan; 2 International Institute for Advanced Studies, Kyoto, Japan; 3 Field Science Education and Research Center, Kyoto University, Kyoto, Japan; 4 Graduate School of Agriculture, Kyoto University, Kyoto, Japan; COMSATS University Islamabad, PAKISTAN

## Abstract

We proposed a simple hyperbolic positioning method that does not require solving simultaneous quadratic equations. Moreover, we introduced the mathematical concept of a “pencil” into analytical calculations in the hyperbolic positioning method for a better understanding. In many recent studies using positioning biotelemetry, the specific procedure for intersection calculation of hyperbolas has rarely been described. This might be one of two major obstacles, with the other being clock synchronisation among receivers, for positioning biotelemetry users, including potential users. We focus only on the intersection calculation in this paper. Therefore, we propose a novel method and introduce the mathematical concept into analytical calculations. The computing performances of the novel method, an analytical method applying the concept of a pencil, and an approximating method using the Newton-Raphson method were compared regarding positioning correctness, accuracy, and calculation speed. In the novel method, hyperbolas were represented using the parameter *θ*, which was treated as a discrete variant. The finer the tick-width of the parameter *θ*, the more accurate was its positioning, but it took slightly longer to calculate. By setting the tick-width to 0.01°, a simulated trajectory was correctly and accurately localised, as in the analytical method which always correctly returned the accurate solution. The approximating method has a major limitation concerning correctness. It returns a single solution regardless of two intersections of hyperbolas; however, the positioning is accurate when the hyperbolas intersect at a single point. This study approached one major difficulty in positioning biotelemetry and will help biotelemetry users overcome this drawback with a simple and intuitive understanding of hyperbolic positioning.

## Introduction

Acoustic biotelemetry techniques have been applied to elucidate the ecology and biology of various free-ranging aquatic animals [1–4]. Biotelemetry systems comprising ultrasonic transmitters and receivers can be used to investigate when and where a tagged animal has been. The position of receivers, which are towed by a boat or placed at a fixed array, and detection time together, record and indicate the position of a tagged animal as a function of time. Spatial resolution of measurement positions could depend upon the density of receivers deployed the target area, by focusing on ‘where’ a tagged animal was. The transmitter and receiver pair possesses a certain range, within which signals can be detected. Detection is affected by the transmitter’s sound pressure level, frequency, and various environmental factors including installation depth and/or surface wind [5]. Arranging receivers without overlapping the detection area can indicate that an animal was at least within the detection area of a certain receiver recording a signal. Deploying receivers with overlapping detection areas enables us to precisely estimate the location of a tagged animal using detection data of the same signal by three or more receivers. One of the simplest localising methods is to adopt weighted centroid method on receivers by recording the same signals for a period as the estimated position [6,7]. The exact positions of animals can be estimated using the time-difference-of-arrival (TDOA) method with a precision of approximately <10 m [8–10]. Several biotelemetry systems have recently achieved the localisation of animals with finer resolutions of approximately <1 m [11–13].

Fine-scale positioning generally employs a hyperbolic positioning method based on TDOA among three fixed receivers [14]. In this method, two hyperbolas are constructed using the receivers’ locations and the difference in distance between the receivers and the sound source, which is calculated from TDOA and sound speed. Then, position of the sound source is determined by finding the intersection of the two hyperbolas. This technique has been used in radio navigation systems such as LORAN-A, LORAN-C, Decca, and omega. A mathematical algorithm of this method on a two dimensional (2-D) plane is briefly presented as follows. Note that we assume that the internal clocks of the receivers are fully synchronised. It is presumed that an acoustic signal is emitted by a particular transmitter. Then, the signal is detected and its arrival times are recorded by three fixed receivers R_1_, R_2_, and R_3_, which are deployed at known locations, at times *t*_1_, *t*_2_ and *t*_3_, respectively. Let *c* be the speed of underwater sound. The positional coordinates of both R_1_ and R_2_, a TDOA *t*_2_—*t*_1_, and *c* define a single branch of a hyperbola H_1_, which is termed as a hyperbolic line of position (LOP) (details in Methods section). It is mathematically known that the transmitter is located somewhere on H_1_, but its exact position is unknown. Similarly, another hyperbolic LOP H_2_ is defined, on which the transmitter is also located. (the other hyperbolic LOP H_3_ can also be defined. However, H_3_ is not always necessary because the intersection is mathematically determined by only one pair of the three hyperbolic LOPs.) Thus, if we can find the intersection of H_1_ and H_2_, then the transmitter is positioned exactly at this intersection. However, this method presents an issue of practical interest, which is finding a concrete way to find that intersection.

The hyperbola is a quadratic curve; therefore, it is necessary to solve simultaneous quadratic equations to find the intersection of curves. There are two ways to solve this issue: analytical and numerical. First, to analytically find the intersection (direct method), we must algebraically solve the simultaneous equations, which is very tedious.- The calculation flow for solving simultaneous quadratic equations has been well described in previous studies using various methods [15–18]. If one uses a computing software, it is possible to solve simultaneous equations by using a ‘solver’ function. If a math-specific programming language such as Matlab (The Math Works, Natick, MA, USA) or Wolfram|Alpha (Wolfram Alpha LLC, IL, USA) is used, the simultaneous equations can be easily solved, although it may increase the cost a little, along with computing time. Alternatively, approximation techniques can be used to numerically determine the intersection. One of the most common methods is the Newton-Raphson method. This method approaches the root(s) of the equation(s) by iterative calculation of the tangential line of a target function. It is a powerful approximation algorithm, especially with regard to the convergent speed if an appropriate initial value is set. This method is implemented through several programming languages such as the nleqslv function in R [19], which makes writing a code for it relatively easy. However, the Newton-Raphson method will not converge if an inappropriate initial value is set, and it does not always return correct solutions if there are multiple roots.

There are many methods to find intersections of hyperbolas, and all of them can potentially provide the same answer. However, the specific procedure for intersection calculation using acoustic positioning biotelemetry has rarely been described previously. Recent papers have vaguely indicated a calculation method without citation [8,9,11–14,20,21]. Calculating the intersection of hyperbolas is a major difficulty that biotelemetry users encounter while obtaining positioning results from acquired receiver data. The analytical (algebraic) way to calculate intersections between two quadric surfaces such as hyperboloids and ellipsoids in a context of the hyperbolic navigation have been described [15,16]. It is mathematically more general than intersection calculation between quadratic curves. However, it is not intuitive because they are not examples of biotelemetry and involves a redundant list of equations. Other papers have illustrated calculation methods in the context of biotelemetry [17,18]. These have been described with only the list of mathematically technical equations, thus it would be essentially the same as the previous set of papers with respect to difficulty associated with intuitive understanding [17]. Furthermore, a calculation method can be used only on a 2-D plane, not in a 3-D space, and has an ambiguous choice of solutions [18]. When solving numerically (approximately) rather than analytically, it may result in the problem of always returning a single answer, even though there are two possible answers, and the calculation method used for this is not clearly stated [8,9,11–14,20,21]. Another obstacle in the positioning procedure, is clock synchronisation among the receivers. However, in this study, we focus only on intersection calculation and not on clock synchronisation because the latter has been well described [14,22]. Therefore, to overcome this major obstacle faced during positioning by biotelemetry users, we aimed (1) to propose a simple and intuitive method for obtaining intersections in a hyperbolic positioning method without solving a simultaneous quadratic equations, (2) to introduce a mathematical concept into analytical (algebraic) calculations for better, consistent understanding; and (3) to compare the positioning performance of this novel method, an analytical (algebraic) method using the introduced concept, and a numerical (approximating) calculation method.

## Methods

### Algorithm of proposed positioning method

An outline of the basic concept of a hyperbolic positioning method has been provided previously. Geometrically, an intersection of a set of hyperbolic LOPs is a transmitter position on a 2-D plane, while in an underwater 3-D space, the intersection of a set of hyperboloids, especially one sheet of a circular hyperboloid of two sheets, and a plane defined by a transmitter depth is the transmitter position. Transmitter depth is obtained if a signal includes depth information (a transmitter has a depth sensor); otherwise, depth should be assumed appropriately, in which case, it might be the same depth as the receivers. Note that we assume that all receivers were deployed at the same depth. One can obtain a hyperbola in the 2-D plane when slicing a hyperboloid by a depth plane. Therefore, we calculate the intersection of a set of hyperbolic LOPs.

The hyperbola is defined by a locus of points *P*(*x*, *y*) such that the difference between the distances from two fixed points *F*_1_(*f*, 0), *F*_2_(-*f*, 0), *f* > 0 (the foci) is a constant, denoted by 2*a*, *a* > 0.


$$\left|PF_{1}-PF_{2}|=2a.\;(1\right)$$

Eq (1) leads to an equation of the hyperbola as follows:

$$\frac{x^{2}}{a^{2}}-\frac{y^{2}}{b^{2}}=1\;(2)$$

$$b^{2}=f^{2}-a^{2},\;(3)$$

where *a* > *b* > 0 and *f* > 0.

While considering acoustic positioning in biotelemetry, let foci *F*_1_ and *F*_2_ be acoustic receivers R_1_ and R_2_, respectively. Assume that an acoustic signal from a particular transmitter at point *P*(*x*, *y*) is detected by the two receivers at *t*_1_ and *t*_2_. Then, the time difference of arrival (TDOA) is |*t*_2_—*t*_1_|. Let *c* be the speed of underwater sound. Let 2*a* be the difference in the distance between the two receivers and the transmitter.


$$c|t_{2}-t_{1}|=2a.\;(4)$$

Eq (4) can be used to define the hyperbola. Therefore, the transmitter will be on a hyperbola which satisfies Eq (4). Parameters *a* and *b* are defined by transforming Eqs (3) and (4). Let *d* be the distance between R_1_ and R_2_, thus,

$$a=\frac{c|t_{2}-t_{1}|}{2}\;(5)$$

$$b=\sqrt{{\left(\frac{d}{2}\right)}^{2}-a^{2}}.\;(6)$$

**Fig 1:**
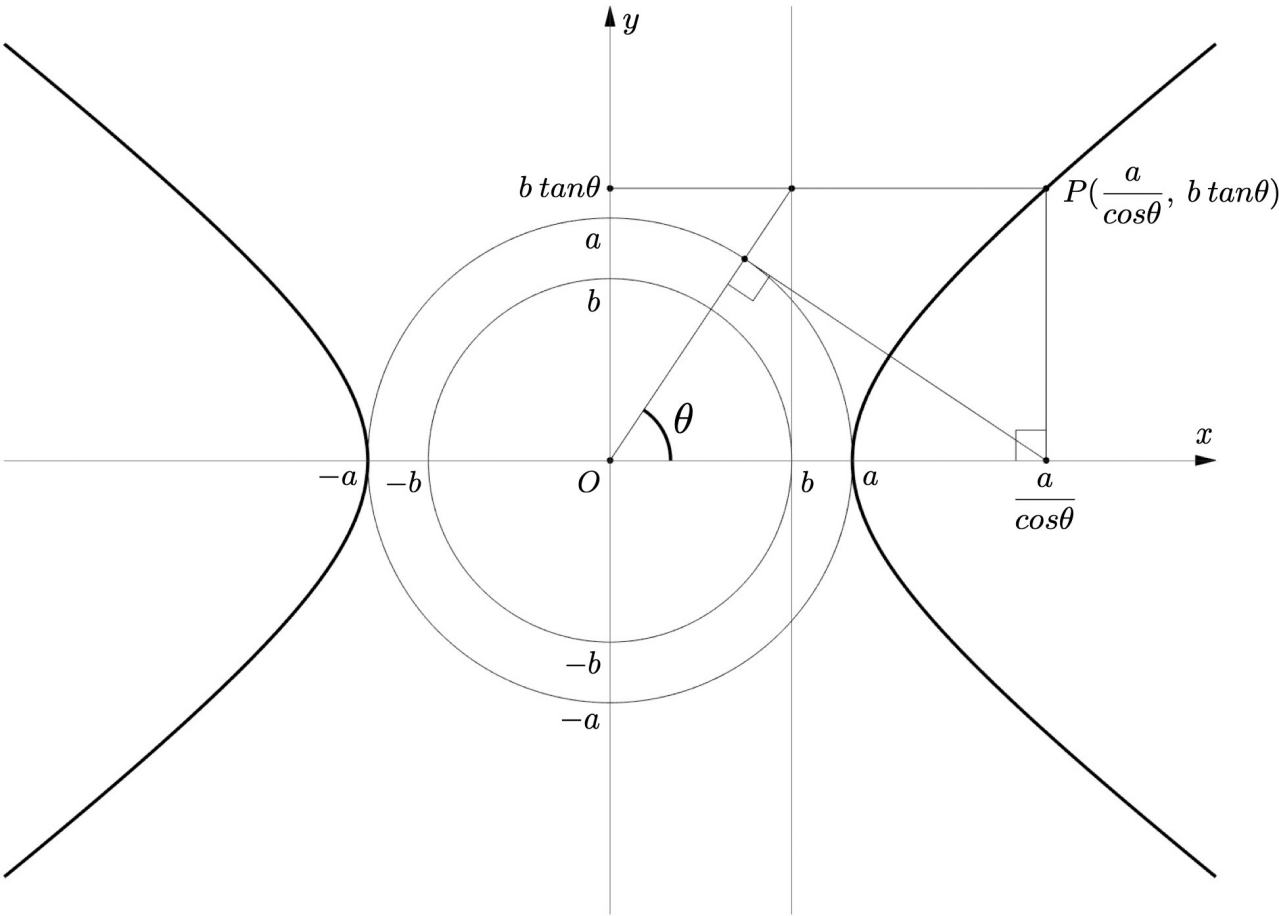
Parameters *a*, *b* (*a* > *b* > 0), and *θ* (-π/2 < *θ <* π/2, π/2 < *θ <* 3π/2) prescribe a hyperbola. A pair of bold lines facing each other represent a hyperbola, which is the locus of *P* uniquely corresponding to *θ*.

A hyperbola can be also represented by introducing a new parameter *θ* (-π/2 < *θ <* π/2, π/2 < *θ <* 3π/2) as follows (Fig 1):

$$\left\{ \begin{array}{c} x=\frac{a}{\cos\;\theta }\;(7)\\ y=b\;\tan\;\theta .\;(8) \end{array}\right.$$

Accordingly, *a* and *b* represent the coordinates of a hyperbola with *θ*.

Here, considering the order of arrival times *t*_1_ and *t*_2_, and the domain of *θ*, only one branch (R1—or R_2_-side branch) of a hyperbola, that is, a hyperbolic LOP, can be defined. In the case of *t*_2_—*t*_1_ > 0, in which R_1_ detected the signal primarily rather than R_2_, meaning that the transmitter would exist closer to R_1_ than to R_2_, so R_1_-side branch should be selected. In the other case of *t*_2_—*t*_1_ < 0, R_2_-side branch can be defined in the same manner. (Note that no hyperbola exists in the case of *t*_2_—*t*_1_ = 0, i.e., a vertical bisector of R_1_R_2_.) This fact is equivalent to considering the sign of Eq (7). Assuming that the domain of *θ* is -π/2 < *θ <* π/2 such that *cosθ* > 0, the sign of *a* prescribes the sign of *x*. Therefore, by letting the sign of *t*_2_—*t*_1_ prescribe the sign of *a*, we redefine *a* as a signed value as follows:

$$a=\frac{c(t_{2}-t_{1})}{2}.\;(9)$$

**Fig 2:**
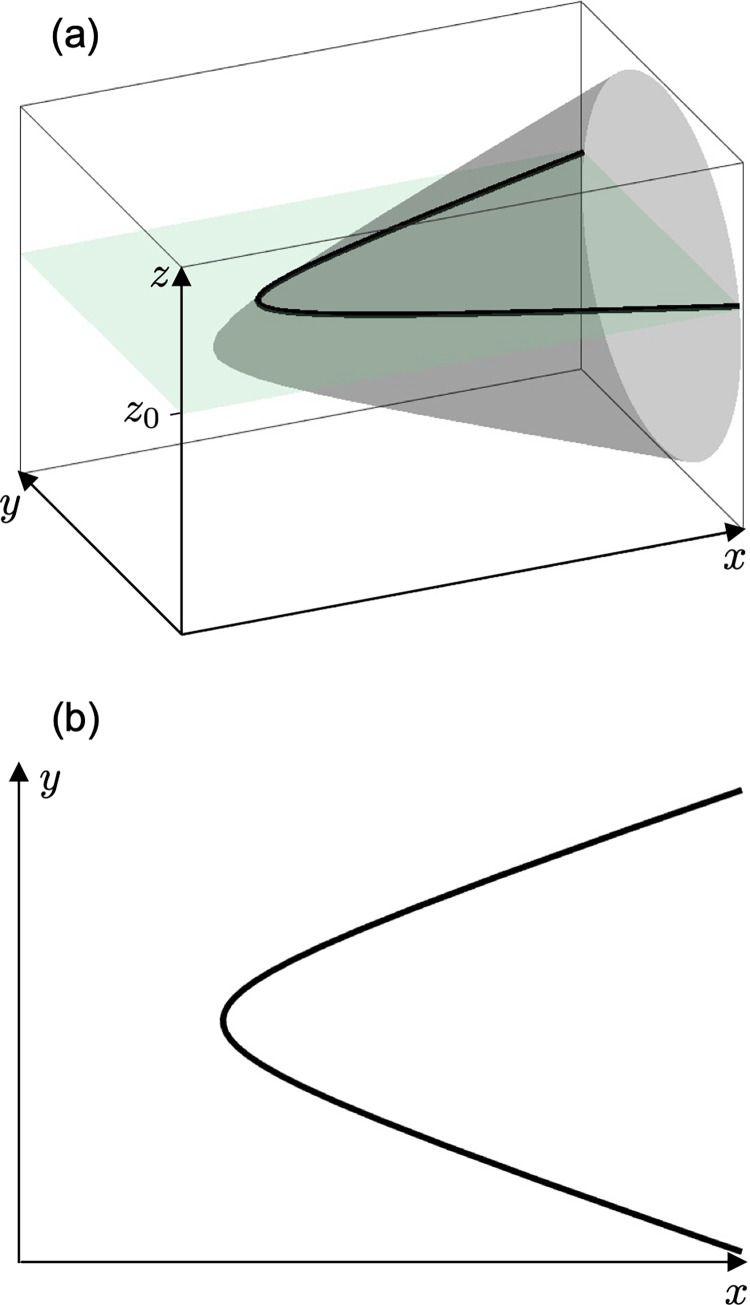
A hyperbola obtained by slicing a circular hyperboloid of two sheets in a plane. (a) The grey hyperboloid is sliced by a light green plane of *z* = *z*_0_. A bold black line represents a hyperbola. (b) A hyperbola on *x*-*y* plane obtained in (a). One side sheet of hyperboloid is drawn as an example.

If transmitter depth is known, a hyperbola can be obtained by slicing a circular hyperboloid of two sheets in a plane of transmitter depth (Fig 2). Parameters *a*’ and *b*’ in this case are defined by multiplying a correction coefficient with *a* and *b*, respectively.

$$a\prime =a\sqrt{1+{\left(\frac{z_{0}}{b}\right)}^{2}}\;(10)$$

$$b^{\prime }=b\sqrt{1+{\left(\frac{z_{0}}{b}\right)}^{2}},\;(11)$$

where *z*_0_ is the absolute difference between receiver installation depth and transmitter depth. Correction coefficient is derived from the equation of a circular hyperboloid of two sheets. S1 File shows the detailed procedure for derivation.

**Fig 3:**
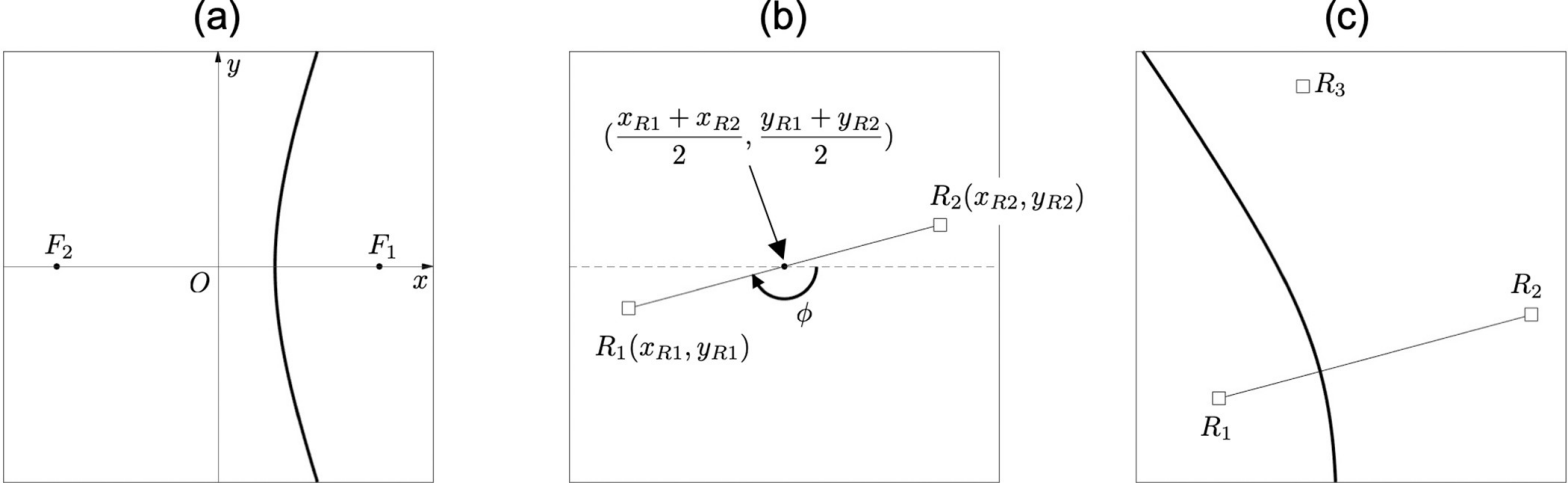
Making a hyperbola in the real coordinate system. (a) A hyperbola (a bold black line) drawn in a coordinate system where the foci *F*_1_ and *F*_2_, i.e., the receivers *R*_1_ and *R*_2_, are on the *x*-axis. (b) Translation distance and rotation angle calculated from *x*-*y* coordinates of receivers in the real coordinate system. The midpoint between receivers represents the translation distance, and *φ* represents rotation angle. (c) A hyperbola in the real coordinate system obtained by rotating and translating a hyperbola in (a).

The hyperbola is drawn on a coordinate system in which the receivers R_1_ and R_2_ are foci on the *x*-axis (Fig 3A). By rotating and translating the foci to match the actual receiver’s position in real coordinates, we can obtain a hyperbola on the real coordinates (Fig 3B and 3C). The translation distances *δx* and *δy* are distances from the origin to the midpoint between actual receiver positions of R_1_(*x*_R1_, *y*_R1_) and R_2_(*x*_R2_, *y*_R2_).


$$\left\{ \begin{array}{c} \delta x=\frac{x_{R1}+x_{R2}}{2}\;(12)\\ \delta y=\frac{y_{R1}+y_{R2}}{2}.\;(13) \end{array}\right.$$

The rotation angle *φ* is an angle formed by *x*-axis and R_1_.


$$\varphi =arctan\frac{y_{R1}-\delta y}{x_{R1}-\delta x}.\;(14)$$

Note that *φ* is easily calculated using *arctan*2 or *atan*2, which is a powerful programming function implemented in almost all programming languages that returns an exact and unique value in the range of -π < *φ* ≤ *π*. For *x* and *y* calculated from Eqs (7) and (8), the coordinates *X* and *Y* given a rotation and translation are as follows:

$$\left\{ \begin{array}{l} X=x\;\cos\;\varphi -y\;\sin\;\varphi +\delta x\;(15)\\ Y=x\;\sin\;\varphi +y\;\cos\;\varphi +\delta y.\;(16) \end{array}\right.$$

**Fig 4:**
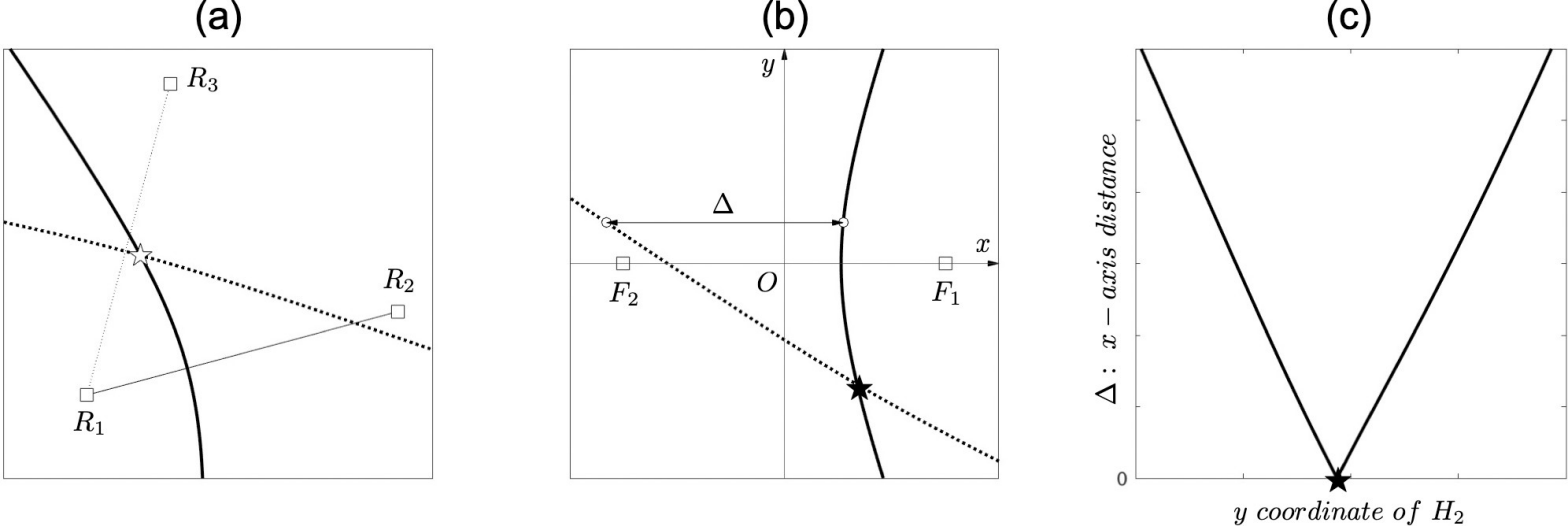
A method for seeking an intersection of two hyperbolas. (a) Two hyperbolas in the Real coordinate system. R_1_, R_2_, and R_3_ represent the receivers. A bold line represents a hyperbola (H_1_) having R_1_ and R_2_ as the foci. A bold dotted line represents a hyperbola (H_2_) having R_1_ and R_3_ as the foci. A white star represents an intersection in the *R*-coordinate system. (b) H_1_ and H_2_ are rotated and translated to fit the H_1_-coordinate system where R_1_ and R_2_ are on *x*-axis as the foci, *F*_1_ and *F*_2_, respectively. A black star represents an intersection in the H_1_-coordinate system. *Δ* is the distance in *x*-axis between H_1_ and H_2_. (c) *Δ* is a function of *y* coordinate of H_2_. There is an intersection at the point of *Δ* = 0. Assuming that H_2_ comprised discrete points, an intersection is at a point where *Δ* is the minimum value.

As described above, we can obtain the hyperbolic LOP H_1_ from the TDOA between R_1_ and R_2_, and those coordinates (Fig 3C). The same procedure for receivers R_1_ and R_3_ allows us to draw another hyperbolic LOP H_2_. The intersection of H_1_ and H_2_ is the position at which the transmitter emitted the signal, that is, the estimated position (Fig 4A).

We shall present our calculation method for the intersection of H_1_ and H_2_. Let *H*_n_- be the coordinate system where the hyperbola before rotation and translation are given. Let the *R*- be the coordinate system where the hyperbola after rotation and translation are given. Let (*x*_n_, *y*_n_) and (*X*_n_, *Y*_n_) be the coordinates of the hyperbola on *H*_n_- and *R*-coordinate systems, respectively. (*X*_n_, *Y*_n_) is a function of (*x*_n_, *y*_n_), rotation angle *φ*_n_, translation distance *δx*_n_ and *δy*_n_, and parameter *θ*. Here, we calculate the intersection of H_1_ and H_2_ on *H*_1_-coordinate system by rotating and translating H_2_(*X*_2_, *Y*_2_). The new coordinates of H_2_’(*x*_2_’, *y*_2_’), which are translated and rotated to fit *H*_1_-coordinate system, are as follows:

$$\left\{ \begin{array}{l} x_{2}\prime =(X_{2}-{\delta x}_{1})\cos\left(-\varphi_{1}\right)-(Y_{2}-{\delta y}_{1})\sin\left(-\varphi_{1}\right)\;(17)\\ y_{2}^{\prime }=(X_{2}-{\delta x}_{1})\sin\left(-\varphi_{1}\right)+(Y_{2}-{\delta y}_{1})\cos\left(-\varphi_{1}\right).\;(18) \end{array}\right.$$

Solving Eq (2) for *x*, we can consider *x* as a function of *y*, meaning that we can represent the coordinate *x*_1_ of H_1_ as a function of given *y* on *H*_1_-coordinate system. Thus, *x*_1_ is defined by *y*_2_’ that is *y* coordinate of H_2_ on *H*_1_-coordinate system.

$$x_{1}=a_{1}\sqrt{1+\frac{{\left(y_{2}\prime \right)}^{2}}{{b_{1}}^{2}}},\;(19)$$

where *a*_1_ and *b*_1_ are derived from Eqs (9) and (6) (or (10) and (11)), respectively. When H_1_ and H_2_ are placed on *H*_1_-coordinate system, there is a unique pair of *x*_1_ and *x*_2_’ for any *y*_2_’. *Δ* is defined as the absolute distance between *x*_1_ and *x*_2_’:

$$\Delta =|x_{1}-x_{2}\prime |.\;(20)$$

**Fig 5:**
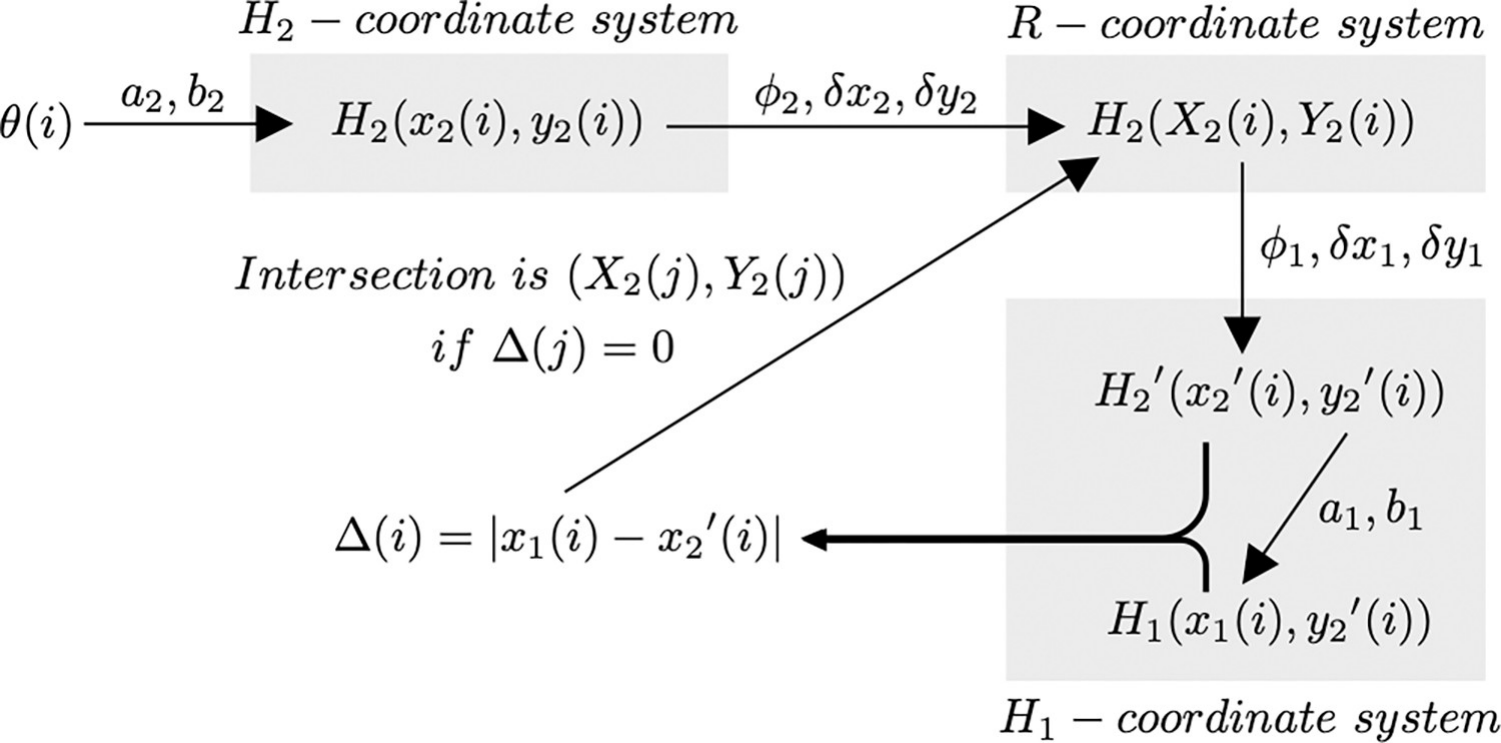
Schematic diagram of the proposed intersection calculation method. There is a consistent correspondence from *θ* to *Δ* via index *i*.

*Δ* represents *x*-axis distance between H_1_ and H_2_ for any *y*_2_’ on *H*_1_-coordinate. Therefore, it is clear that H_1_ and H_2_ must intersect at the point where *Δ* = 0 (Fig 4B and 4C). Further, everything from hyperbolic LOP (*x*_n_, *y*_n_) to *Δ* is consistently a function of *θ*. If, *θ* is treated as a discrete sequence, there is a consistent correspondence between *θ* and *Δ* via index *i* from *θ* to *Δ* (Fig 5). Hence, seeking an index *j* that suffices *Δ*(*j*) = 0, or *Δ*(*j*) ≈ 0, is equal to seeking the intersection of H_1_ and H_2_. Thus, the intersection on the *R*-coordinate system, which we want to find, is (*X*_2_(*j*), *Y*_2_(*j*)).

### Analytical solution

Simultaneous quadratic equations must be solved to analytically determine the intersection of hyperbolas. As it is a troublesome task, we introduce a mathematical concept of a ‘pencil’ to help understand this calculation procedure. When the pencil is applied to the intersection calculation of two quadratic curves, finding intersections of the two curves is transformed into finding intersections of one of the curves with straight lines, which are generated from the two curves. Let both *f*(*x*, *y*) = 0 and *g*(*x*, *y*) = 0 be quadratic curves, and assuming that those curves intersect at a point of (*s*, *t*). Then,

f(s,t)=0∧g(s,t)=0(21)

**Fig 6:**
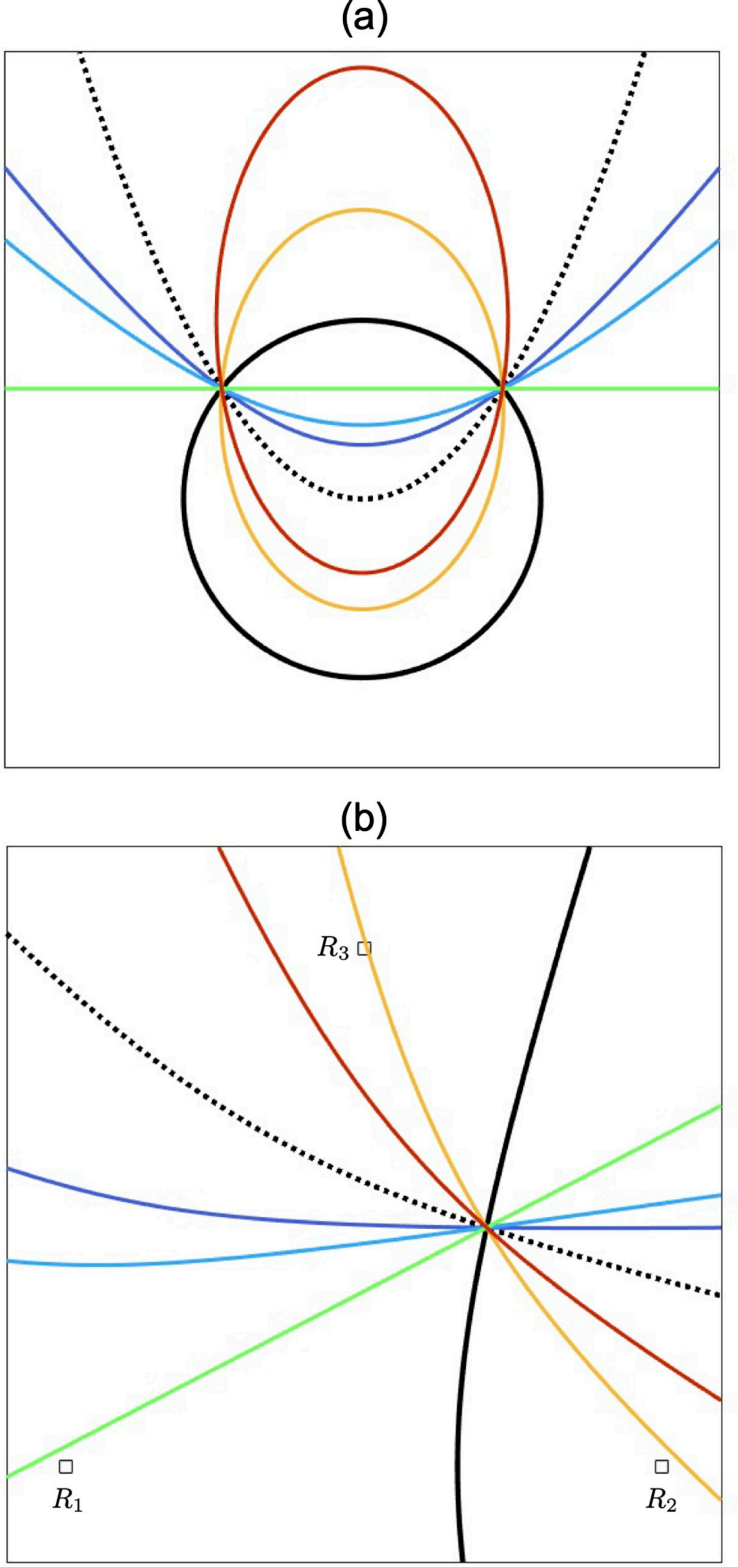
Examples for a pencil made from two quadratic curves. (a) A pencil made from a circle (a black line) and a parabola (a black dotted line). Coloured lines indicate a set of quadratic curves, i.e., a pencil. All curves pass through the two intersections of the original circle with the original parabola. The concrete equations are as follows: The circle is *f*(*x*, *y*) = *x*^2^ + *y*^2^–1 = 0, the parabola is *g*(*x*, *y*) = *x*^2^—*y* = 0, and the pencil is *f*(*x*, *y*) + *λg*(*x*, *y*) = 0, where *λ =* -3, -2, -1, 1, 2 (∈ ℝ). There is a straight line (a green line) when *λ =* -1. If *λ* = 0, the curve is the same as the original circle. (b) A pencil made from two hyperbolic LOPs. White squares are foci of the hyperbolic LOPs, i.e., the receivers. A black curve represents a hyperbolic LOP having foci of R_1_ and R_2_, and a black dotted curve represents a hyperbolic LOP having foci of R_1_ and R_3_. Coloured lines indicate a set of quadratic curves, i.e., a pencil. All curves pass through the intersection of two hyperbolic LOPs. The concrete equations are as follows: The black line hyperbolic LOP is $f(x,y)=200x-63\sqrt{x^{2}+y^{2}}-9007.75=0$, the black dotted line hyperbolic LOP is $g(x,y)=100x+174y-60\sqrt{x^{2}+y^{2}}-9169=0$, and the pencil is *f*(*x*, *y*) + *λg*(*x*, *y*) = 0, where *λ =* -3, -2, -1, 1, 2 (∈ ℝ). There is a straight line (a green line) when *λ =* -1. If *λ =* 0, the curve is the same as the black line hyperbolic LOP.

⟺f(s,t)+λg(s,t)=0,(22)

where *λ* is any real number. This means Eq (22) always passes through (*s*, *t*) regardless of the value of *λ*. Therefore, *f*(*x*, *y*) + *λg*(*x*, *y*) = 0 is a set of quadratic curves including a line passing through (*s*, *t*), which is an intersection of *f*(*x*, *y*) = 0 and *g*(*x*, *y*) = 0 (Fig 6A). This is a brief description of using the concept of pencil.

**Fig 7:**
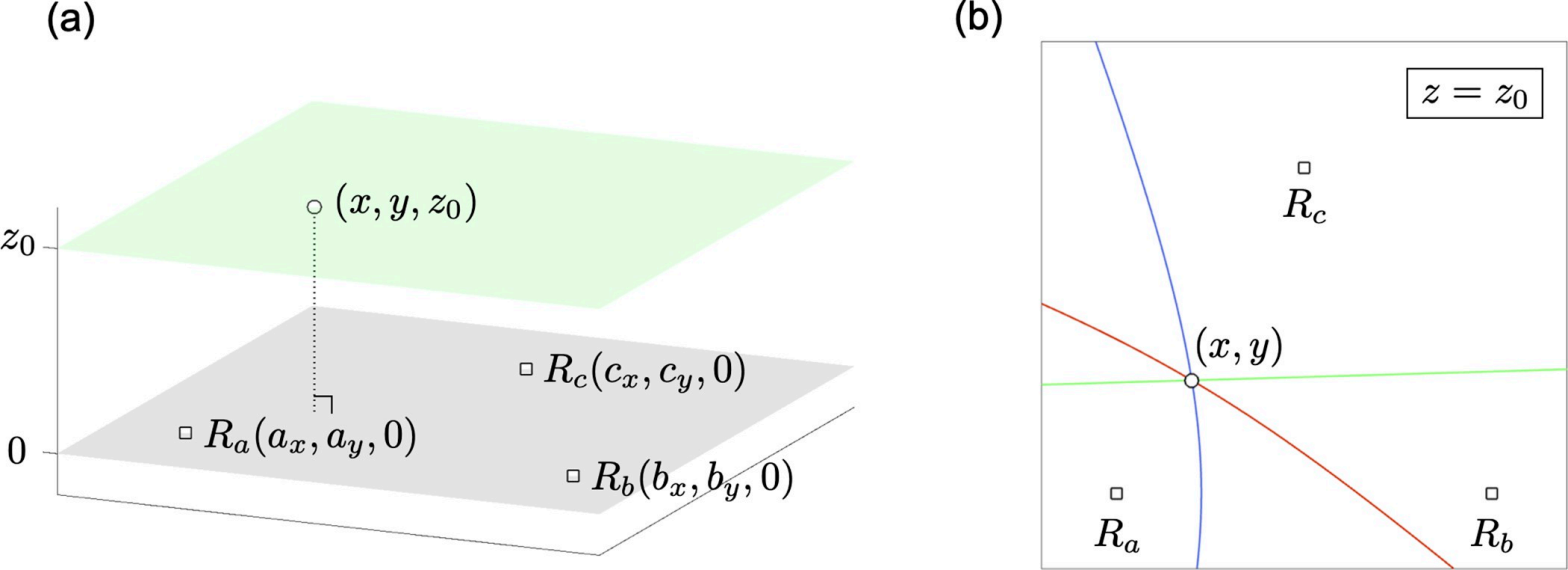
Image of intersection calculation of two hyperbolic LOPs in positioning biotelemetry by applying a pencil. (a) 3-D position of three receivers (*R*_*a*_, *R*_*b*_, and *R*_*c*_: white square) and a transmitter (white circle). For convenience, the receivers are deployed on the plane of *z = 0* (gray plane), and the transmitter on the plane of *z = z*_*0*_ (green plane). Note that *z*_*0*_ is not transmitter depth, but absolute difference between transmitter depth and the receivers’ installation depth. (b) Two hyperbolic LOPs (blue and red curves) and a straight line (green line) generated from those two hyperbolic LOPs by applying a pencil on the plane of *z = z*_*0*_. Note that the receivers are not foci of the hyperbolas on the plane except for *z*_*0*_ *= 0*.

The specific procedure for intersection calculation of two hyperbolas in positioning biotelemetry is as follows. An acoustic signal emitted by a transmitter is detected by three fixed receivers *R*_*a*_(*a*_*x*_, *a*_*y*_, 0), *R*_*b*_(*b*_*x*_, *b*_*y*_, 0), and *R*_*c*_(*c*_*x*_, *c*_*y*_, 0) at time *t*_*a*_, *t*_*b*_ and *t*_*c*_, respectively (Fig 7A). Note that the receivers are assumed to be deployed at the same depth. The TDOA between *R*_*a*_ and *R*_*b*_, and *R*_*a*_ and *R*_*c*_ are *T*_*ab*_ = *t*_*a*_—*t*_*b*_, and *T*_*ac*_ = *t*_*a*_—*t*_*c*_, respectively. Let *c* be the underwater speed of the sound. The position of a transmitter (*x*, *y*, *z*) suffices following equations:

$$\left\{ \begin{array}{l} \sqrt{{\left(x-a_{x}\right)}^{2}+{\left(y-a_{y}\right)}^{2}+z^{2}}-\sqrt{{\left(x-b_{x}\right)}^{2}+{\left(y-b_{y}\right)}^{2}+z^{2}}=R_{ab}\;(23)\\ \sqrt{{\left(x-a_{x}\right)}^{2}+{\left(y-a_{y}\right)}^{2}+z^{2}}-\sqrt{{\left(x-c_{x}\right)}^{2}+{\left(y-c_{y}\right)}^{2}+z^{2}}=R_{ac},\;(24) \end{array}\right.$$

where,

$$\begin{array}{c} R_{ab}=cT_{ab}\\ R_{ac}=cT_{ac}. \end{array}$$

These equations provide the definition of a hyperbola. Transposing the first term of the left-hand side to the right-hand side and squaring the two sides, we obtain

$$\left\{ \begin{array}{l} 2(b_{x}-a_{x})x+2(b_{y}-a_{y})y-2R_{ab}\sqrt{{\left(x-a_{x}\right)}^{2}+{\left(y-a_{y}\right)}^{2}+z^{2}}+{R_{ab}}^{2}+A-B=0\;(25)\\ 2(c_{x}-a_{x})x+2(c_{y}-a_{y})y-2R_{ac}\sqrt{{\left(x-a_{x}\right)}^{2}+{\left(y-a_{y}\right)}^{2}+z^{2}}+{R_{ac}}^{2}+A-C=0,\;(26) \end{array}\right.$$

where,

$$A={a_{x}}^{2}+{a_{y}}^{2}$$

$$B={b_{x}}^{2}+{b_{y}}^{2}$$

$$C={c_{x}}^{2}+{c_{y}}^{2}.$$

Eqs (23) and (24) are equivalent to (25) and (26), respectively, which represent the same hyperbolic LOPs (Fig 7B). Note that if Eqs (25) and (26) are transformed by squaring to omit the roots, the new equations represent hyperbolas. By applying the concept of a pencil, a straight line can be generated from Eqs (25) and (26) (Fig 7B). Considering that Eqs (25) and (26) are *f*(*x*, *y*) = 0 and *g*(*x*, *y*) = 0, respectively. We need to find the value of *λ* such that the equation *f*(*x*, *y*) + *λg*(*x*, *y*) = 0 is linear, that is,

$$\lambda =-\frac{R_{ab}}{R_{ac}}.\;(27)$$

Substituting this *λ* into *f*(*x*, *y*) + *λg*(*x*, *y*) = 0 and solving it, we obtain the following equation for a straight line.

y=Dx+E,(28)

where,

$$D=\frac{R_{ab}(c_{x}-a_{x})-R_{ac}(b_{x}-a_{x})}{R_{ac}(b_{y}-a_{y})-R_{ab}(c_{y}-a_{y})}$$

$$E=\frac{R_{ab}({R_{ac}}^{2}+A-C)-R_{ac}({R_{ab}}^{2}+A-B)}{2(R_{ac}(b_{y}-a_{y})-R_{ab}(c_{y}-a_{y}))}.$$

The intersection of Eqs (25) and (26) is equivalent to the intersection of Eqs (25) and (28) (Fig 7B). Substituting Eqs (28) into (25) and solving it, we obtain

$$Fx^{2}+Gx+(H-z^{2})=0,\;(29)$$

where,

$$F={\left(\frac{I}{R_{ab}}\right)}^{2}-D^{2}-1$$

$$G=\frac{IJ}{{R_{ab}}^{2}}-2(D(E-a_{y})-a_{x})$$

$$H={\left(\frac{J}{2R_{ab}}\right)}^{2}-A-E^{2}+2a_{y}E$$

$$I=b_{x}-a_{x}+D(b_{y}-a_{y})$$

$$J=2E(b_{y}-a_{y})+{R_{ab}}^{2}+A-B.$$

If the depth of transmitter is known as *z*_0_, that is, the transmitter has a depth sensor, substituting *z* = *z*_0_ with Eq (29); otherwise, use an appropriate value, which might be zero in most cases, to *z*_0_. If the installation depth of the receivers is not zero, which is a natural situation, *z*_0_ is the absolute difference between the installation depth and the transmitter depth. Applying the quadratic formula to Eq (29) to find *x*, we obtain

$$x=\frac{-G\pm \sqrt{G^{2}-4F(H-{z_{0}}^{2})}}{2F}.\;(30)$$

Then, substituting *x* into Eq (28) to find *y*, two candidates for the intersection are obtained. If there is only one intersection, one of the two candidates should be selected after considering the condition. For example, substituting *x* and *y* in Eq (23), the pair of *x* and *y* that holds equality is the coordinate of the intersection. Where there are two intersections, both pair of *x* and *y* pairs hold equality. In the other case where there is no intersection, *x* and *y* become imaginary numbers.

### Comparison of positioning results

We compared the positioning results of a simulated trajectory using the proposed method, analytical method, and approximation method. A simulated trajectory comprising 200 points was constructed in a 3-D space as it passed through an array of three receivers. The array formed an equilateral triangle of 50 m on each side. Installation depths of the three receivers were set at the same depth of 15 m. It was assumed that the signal, including depth information from a transmitter on the trajectory propagated at the underwater sound speed of 1500 m/s, and that it was detected by all three receivers. There were no misdetections or multipath effects; thus, all data would be correctly calculated. The comparison items are as follows: (1) the number of false answers, i.e., whether the number of solutions is correctly returned, (2) positioning accuracy, and (3) computation time. For (1), the analytical method is treated as it always returns true solutions. For (2), accuracy was the average distance from the true position and the estimated position only when the hyperbolas intersected at one point. For (3), it was expressed as a ratio to the average of 200 calculations using the analytical method, to clearly show differences between the methods, as actual calculation period may highly depend upon the machine power of a computer used. The proposed method was set to three types, with *θ* tick width of 1°, 0.1°, and 0.01°. The analytical method was established by applying the concept of a pencil. An approximation method was constructed using the Newton-Raphson method. The initial coordinates were set at the centroid of the receivers. Iterations were terminated when the difference between the *x*- and *y*-coordinates in the update was <0.001, or when the number of iterations was >5. All the procedures of the three methods were coded in R ver. 4.0.5 [23]. The source codes of the three methods and coordinates of the trajectory are shown in S2 and S3 Files. Computing was performed on a laptop computer of MacBook Pro (13-inch, 2017, Four Thunderbolt 3 Ports) with a processor of 3.1 GHz and 8 GB random-access memory.

## Results

**Fig 8:**
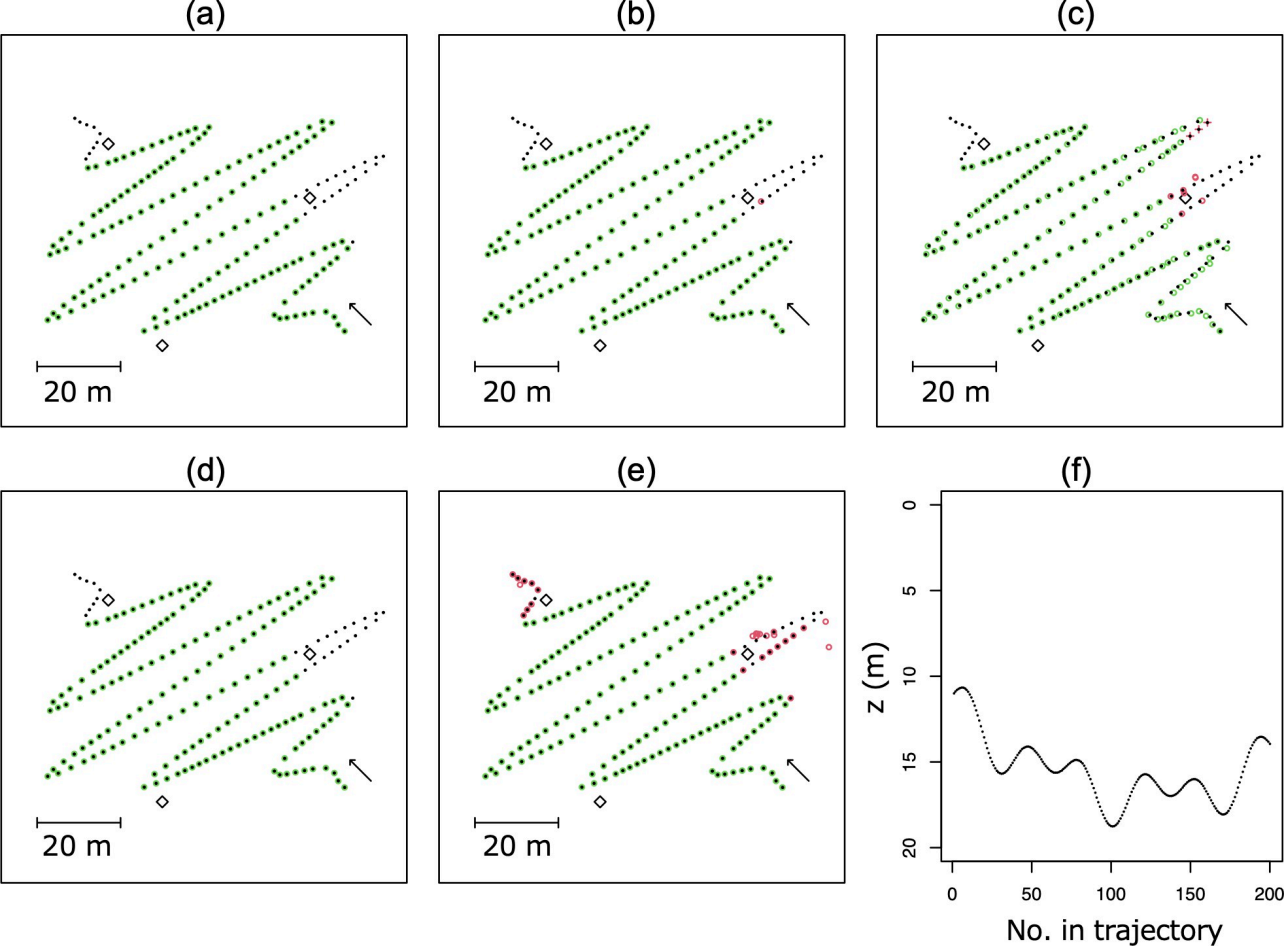
2-D plot of calculation results of the simulated trajectory for the three positioning methods (a–e). White squares represent receivers. Black points indicate true positions of the trajectory. Green circle indicates a calculated position for correct solutions. Red circle indicates the same for incorrect solutions. Red cross indicates that no solution was returned. The arrow represents a direction of the simulated trajectory. (a) Results of the proposed method with *θ* tick width of 0.01°. (b) Results of the proposed method with *θ* tick width of 0.1°. (c) Results of the proposed method with *θ* tick width of 1°. (d) Results of the analytical method. which are the true results of positioning. (e) Results of the approximating method using Newton-Laphson method. (f) *z*-coordinates of the trajectories.

The five computing results of the three methods are graphically presented in Fig 8. The analytical method had no false answer with positioning accuracy of zero (Fig 8E and Table 1). Similar to the analytical method, the proposed method with *θ* tick width of 0.01° also had no false answer with approximately zero accuracy (Fig 8A and Table 1). The proposed method with 0.1° had only one false answer (Fig 8B and Table 1), and that with 1° *θ* tick width had ten false answers including three returns with no answer where there was a single intersection (Fig 8C and Table 1). Positioning accuracy of the proposed method got lower as *θ* tick width got larger (Table 1). The approximating method had 29 false answers, meaning this method always returned single answer even though there were two intersections (Fig 8E). However, positioning accuracy of this method was zero in the case that there was only one intersection (Table 1). Computing period of the analytical method was 3.81 × 10^−4^ sec in average (n = 200). In comparison, the average computing period of other methods ranged for 0.89–9.05 times (Table 1).

**Table 1:** Computing results of three positioning methods.

Methods	*θ* Tick width (°)	Number of false answers	Positioning accuracy (m)	Average computing period (n = 200) ^a^
Proposed method	1	10	0.22 ± 0.21 (0.00–0.98; n = 168)	1.37 ± 16.76 (0.10–237.16)
	0.1	1	0.02 ± 0.02 (0.00–0.11; n = 171)	0.89 ± 3.20 (0.34–38.12)
	0.01	0	0.00 ± 0.00 (0.00–0.01; n = 171)	9.05 ± 7.57 (4.97–43.96)
Analytical method	–	0	0 ± 0 (n = 171)	1
Approximating method	–	29	0.00 ± 0.00 (0.00–0.00; n = 171)	1.63 ± 18.39 (0.21–260.45)

^a^ Average computing period was represented as the ratio to that of the analytical method, 3.81 × 10^−4^ sec (1.00 × 10^−5^–7.37 × 10^−2^).

## Discussion

We proposed a simple, intuitive method and introduced a mathematical concept for intersection calculation in hyperbolic positioning. The calculation procedure in acoustic positioning biotelemetry would have two difficulties in obtaining the positions of target animals: clock synchronisation among receivers and intersection calculation between hyperbolas. A brief explanation of the clock synchronisation technique has been described [14,22], so we focused on intersection calculation in this study. The clock can be synchronised by linear regression using detection data recorded in receivers [14,22]. However, it is slightly more difficult to obtain positions by intersection calculation because of the need to solve simultaneous quadratic equations. This task for finding intersections could be a great obstacle for calculating positioning by biotelemetry users, including potential users. Therefore, we focused on intersection calculation methods.

**Fig 9:**
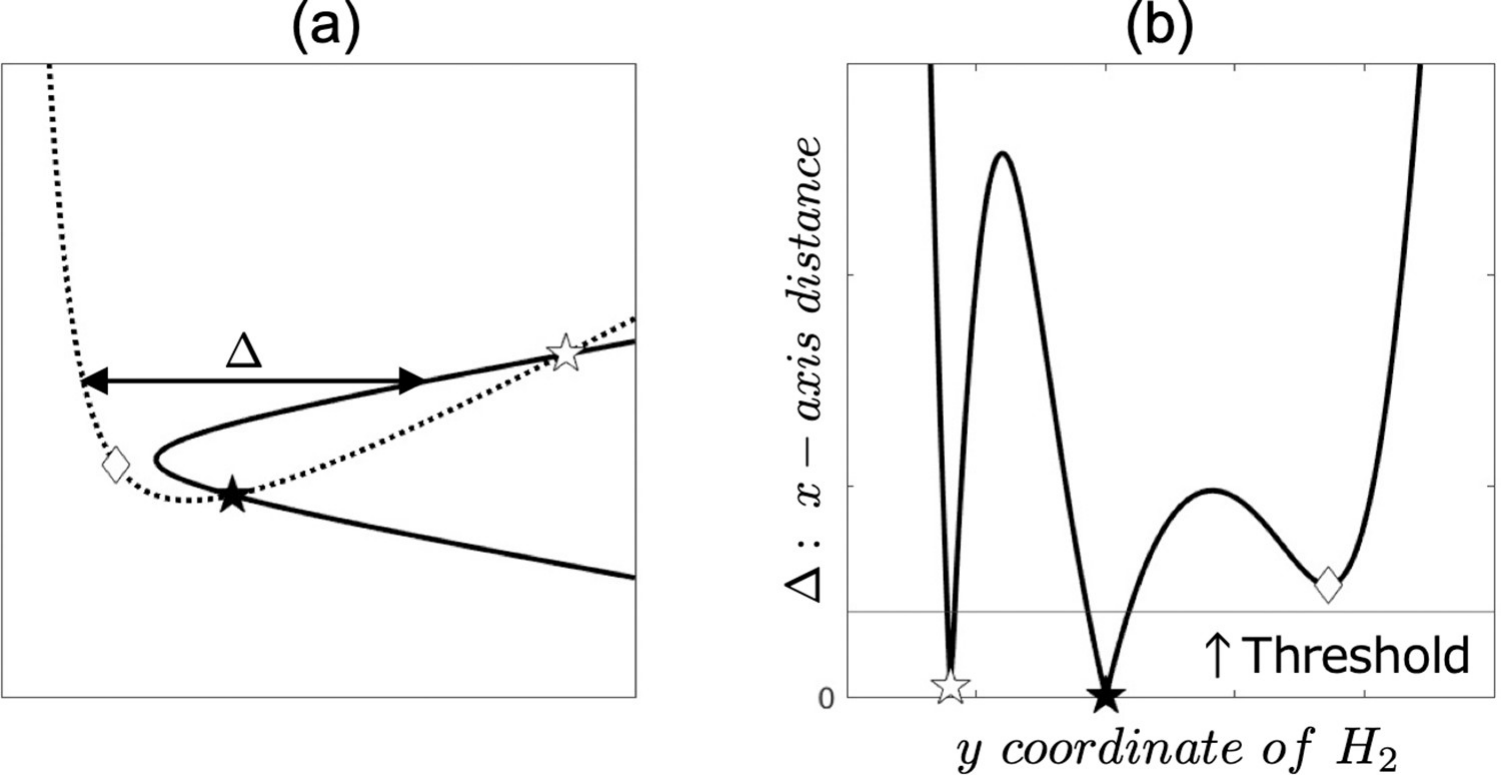
The case of two intersections with three local minimums. (a) *Δ* represents *x*-axis distance between two hyperbolic LOPs (a black curve and a black dotted curve). The hyperbolic LOPs crosses at two points of a white and a black star. (b) *Δ* has three local minimums, two of which indicate intersections. The white and the black star, and the white diamond at each local minimum value of *Δ* corresponds to each symbol in (a). *Δ* showing that the intersections can be selected by using a threshold or considering the order of the local minimum value.

From the computing results for the positioning of the simulated trajectory, it was found that the proposed method with *θ* tick width of 0.01° was able to localise the trajectory with an accuracy of zero m without any false answers, including the analytical method (Fig 8A–8E). The proposed method with *θ* tick width of 1° had several false answers and noticeable errors (Fig 8C). The method with *θ* tick width of 0.1° had a higher accuracy, but one false answer was given (Fig 8D). When the returned answer was incorrect, it was because the shape of a hyperbola could only be roughly drawn owing to the resolution of *θ*. If there were two intersections and the method returned only one solution, the two intersections were too close together to be distinguished. If there was a unique solution but no solution was returned, that possible solution was eliminated because a local minimum of *Δ* was larger than the threshold that was set to omit false intersections (Fig 9B). There was no false answer with *θ* tick width of 0.01°; however, computing period increased considerably as the tick width became finer, owing to the larger length of the variables handled. Although the computing period should be short, it took an average of nine times longer than 3.81 × 10^−4^ s, which was on the order of 10^−3^ s. Although the period depends largely on machine specifications of the computer used for the calculations, the computer used in this study was a laptop, which is commonly available on the market. Therefore, the computing period is unlikely to be a major bottleneck. For the approximating method, there was a serious limitation regarding false answers, that is, the method always returned a single solution even if there were two intersections of hyperbolas. This is a weakness of approximating methods, including the Newton-Raphson method. The approximating method is relatively easy to code using existing functions (e.g., the nleqslv function in R [19]); therefore, special care should be taken when using it. Note that the positioning error is almost zero within <5 iterations (Fig 8B), indicating that the convergence of this method is very fast. This is a strong point of the approximation method using the Newton-Raphson method.

Our proposed method is a little complicated because it employs parameter *θ* to express coordinates of a hyperbola. However, although the equations may seem slightly complicated as they involve trigonometric functions, the operation is essentially simple since it only requires rotations and translations. We drew enough figures to make it intuitive and easy to understand. The proposed method can simply find intersections during position calculation without the requirement to solve quadratic equations. We employed two ideas for the calculation: parameter *θ*, signed *a*. First, parameter *θ* enables us to cover the possible range of a hyperbola. We can also obtain the *x* coordinate of a hyperbola using Eq (19) in the *H*_*n*_-coordinate system. However, considering the possible range of a hyperbola by Eq (19), *y* must range from -*inf* to *inf*, which cannot be represented using programming languages. (Note that *x*_1_ is calculated directly from *y*_2_’ in Eq (19) for calculating *Δ*, but this does not matter here because *y*_2_’ has already been finitely determined by parameter *θ*.) A larger range of *y* would simply require a larger computational complexity if the tick value is consistent. Although it might be possible to apply an optimal range of *y* for each case, this method would not be simple. Parameter *θ* is convenient because we do not have to consider it. Second, the signed *a* enables us to omit the case classification when defining a hyperbolic LOP. Generally, *a* is larger than 0 because *a* is the distance from the origin to the vertex of a hyperbola. In the case of *a* > 0, we must choose one branch of a hyperbola (e.g., R_1_- or R_2_-side branch) based on the order of arrival times. However, integrating the order of arrival times into *a* plus-minus sign would simply and uniquely define which branch is a hyperbolic LOP.

The proposed method can detect an index when there are two intersections of hyperbolic LOPs if the tick width of *θ* is adequate (Fig 8). To find an index *j* that suffices *Δ*(*j*) = 0, we can explore the raw *Δ* value without using a technique such as differentiating Eq (20) by *x*_*2*_’ because *Δ* comprises an array of discretised values. However, due to the discretisation, there is not always an index *j* where *Δ*(*j*) = 0. In addition, there are theoretically one or two intersection points. For these two reasons, it is convenient to consider the local minimum value instead of the minimum value or zero. Although there can be a case where three indices of local minimal value exist, we can omit the third index that does not detect an intersection by some threshold or the order of local minimal values, because intersections must theoretically be up to two points (Fig 9). Practically, the existence of two intersections means that it is impossible to provide a unique position unless a unique position is calculated from another combination of three receivers in the array.

We introduced the mathematical concept of a pencil to help with understanding the calculation procedure intuitively and graphically for hyperbolic positioning. The listed calculation procedure using the equations is approximately similar to when simultaneous equations of two hyperbolic LOPs are solved [15]. However, rather than simply listing the preceding equations, we have tried to present them in a way that makes what is being calculated here, explicit, by employing a pencil. We have also attempted to introduce a pencil so that it can be applied in the future. The concept of a pencil is a powerful tool for finding a sound source for acoustic positioning biotelemetry. Although it is assumed that installation depth of the three receivers is the same in the intersection calculation described in this study, this technique can be applied to different positioning cases. Such cases include, 3-D positioning using four receivers without depth information of a transmitter. The calculation procedure in each case is highly redundant, therefore all of it has not been discussed here; it has been described in S4 File. In positioning biotelemetry, fine-scale positioning of <1 m order will be one trend to observe inter-individual or inter-specific interactions between animals (e.g. [12,24]). Positioning results are geometrically dependent upon coordinates of the receivers, including installation depth, and are particularly sensitive in the vicinity of the receiver because of the shape of a hyperbola or hyperboloid. Mathematically rigorous methods of intersection calculations under various conditions are important in this area.

## Conclusions

This study aims to achieve two goals: proposing a novel positioning method which does not require solving simultaneous quadratic equations, and introducing a mathematical concept of a ‘pencil’ to an analytical (algebraic) positioning method. First, it was found that our proposed method has potential to become a new method in positioning biotelemetry, so far as the tick width remains adequately fine. From the results of positioning simulation, our proposed positioning method could accurately obtain the intersection between hyperbolic LOPs with no false return similar to the analytical method, which is mathematically correct, if tick width of parameter *θ* was ≤0.01°. In contrast, Newton-Raphson method, and our proposed method without fine tick width found some false answers that come from approximating methods, especially in Newton-Raphson method. Second, it was also proved through positioning simulation that an analytical method could be constructed by introducing the concept of a ‘pencil,’ and it could correctly calculate an intersection in positioning biotelemetry. We proved that the analytical method constructed using the concept of a pencil is highly applicable, and that it can be applied to the more complex case of positioning biotelemetry such as calculating 3-D positioning using four receivers as presented in S4 File. However, approximately identical results will be obtained by all methods i.e., the analytical method, proposed method, and approximating method used in this study, although this scenario is only in the case that there is single intersection (i.e., excluding false answers caused by approximating way), because all methods are constructed based on mathematics. Nevertheless, seeking the intersection of hyperbolas may be a major obstacle for calculating positioning by biotelemetry users, including potential users. We tried to overcome this obstacle by enabling the users to solve this issue through a simple and intuitive method that we proposed and introduced in this paper, with a better understanding, rather than through troublesome tasks.

## Supporting information

S1 File Detailed derivation procedure of slicing a hyperbola from a circular hyperboloid of two sheets. (PDF)

S2 File Source code of the three methods for positioning. (PDF)

S3 File 3-D (*x,y,z*) coordinate of the trajectory consisting of 200 points used in the simulation. (CSV)

S4 File Two examples of the mathematical procedure of 3-D positioning by applying the concept of a pencil. (PDF)

## References

[1] Hussey NE, Kessel ST, Aarestrup K, Cooke SJ, Cowley PD, Fisk AT, et al. Ecology. Aquatic animal telemetry: a panoramic window into the underwater world. Science. 2015;348: 1255642. doi: 10.1126/science.1255642 26068859

[2] Hays GC, Ferreira LC, Sequeira AMM, Meekan MG, Duarte CM, Bailey H, et al. Key questions in marine megafauna movement ecology. Trends Ecol Evol. 2016;31: 463–475. doi: 10.1016/j.tree.2016.02.015 26979550

[3] Crossin GT, Heupel MR, Holbrook CM, Hussey NE, Lowerre‐Barbieri SK, Nguyen VM, et al. Acoustic telemetry and fisheries management. Ecol Appl. 2017;27: 1031–1049. doi: 10.1002/eap.1533 28295789

[4] Matley JK, Klinard NV, Barbosa Martins AP, Aarestrup K, Aspillaga E, Cooke SJ, et al. Global trends in aquatic animal tracking with acoustic telemetry. Trends Ecol Evol. 2022;37: 79–94. doi: 10.1016/j.tree.2021.09.001 34563403

[5] Gjelland KØ, Hedger RD. Environmental influence on transmitter detection probability in biotelemetry: developing a general model of acoustic transmission. Methods Ecol Evol. 2013;4: 665–674. doi: 10.1111/2041-210X.12057

[6] Simpfendorfer CA, Heupel MR, Hueter RE. Estimation of short-term centers of activity from an array of omnidirectional hydrophones and its use in studying animal movements. Can J Fish Aquat Sci. 2002;59: 23–32. doi: 10.1139/f01-191

[7] Simpfendorfer CA, Heupel MR, Collins AB. Variation in the performance of acoustic receivers and its implication for positioning algorithms in a riverine setting. Can J Fish Aquat Sci. 2008;65: 482–492. doi: 10.1139/f07-180

[8] Andrews KS, Tolimieri N, Williams GD, Samhouri JF, Harvey CJ, Levin PS. Comparison of fine-scale acoustic monitoring systems using home range size of a demersal fish. Mar Biol. 2011;158: 2377–2387. doi: 10.1007/s00227-011-1724-5

[9] Espinoza M, Farrugia TJ, Webber DM, Smith F, Lowe CG. Testing a new acoustic telemetry technique to quantify long-term, fine-scale movements of aquatic animals. Fish Res. 2011;108: 364–371. doi: 10.1016/j.fishres.2011.01.011

[10] Roy R, Beguin J, Argillier C, Tissot L, Smith F, Smedbol S, et al. Testing the VEMCO Positioning System: spatial distribution of the probability of location and the positioning error in a reservoir. Anim Biotelem. 2014;2: 1–7. doi: 10.1186/2050-3385-2-1

[11] Guzzo MM, Van Leeuwen TE, Hollins J, Koeck B, Newton M, Webber DM, et al. Field testing a novel high residence positioning system for monitoring the fine‐scale movements of aquatic organisms. Methods Ecol Evol. 2018;9: 1478–1488. doi: 10.1111/2041-210X.12993 30008993 PMC6033000

[12] Leclercq E, Zerafa B, Brooker AJ, Davie A, Migaud H. Application of passive-acoustic telemetry to explore the behaviour of ballan wrasse (*Labrus bergylta*) and lumpfish (*Cyclopterus lumpus*) in commercial Scottish salmon sea-pens. Aquaculture. 2018;495: 1–12. doi: 10.1016/j.aquaculture.2018.05.024

[13] Takagi J, Ichikawa K, Arai N, Miyamoto Y, Uchida K, Shoji J, et al. Simultaneous observation of intermittent locomotion of multiple fish by fine-scale spatiotemporal three-dimensional positioning. PLOS ONE. 2018;13: e0201029. doi: 10.1371/journal.pone.0201029 30024958 PMC6053229

[14] Smith F. Understanding HPE in the VEMCO Positioning System (VPS). 2013. [Cited 8 Aug 2022]. Available from: https://www.oceans-research.com/wp-content/uploads/2016/09/understanding-hpe-vps.pdf.

[15] Fang BT. Simple solutions for hyperbolic and related position fixes. IEEE Trans Aerosp Electron Syst. 1990;26: 748–753. doi: 10.1109/7.102710

[16] Bucher R, Misra D. A synthesizable VHDL model of the exact solution for three-dimensional hyperbolic positioning system. VLSI Des. 2002;15: 507–520. doi: 10.1080/1065514021000012129

[17] Deng ZD, Weiland MA, Fu T, Seim TA, LaMarche BL, Choi EY, et al. A cabled acoustic telemetry system for detecting and tracking juvenile salmon: Part 2. Three-dimensional tracking and passage outcomes. Sensors (Basel). 2011;11: 5661–5676. doi: 10.3390/s110605661 22163919 PMC3231425

[18] Lagardère J-P, Ducamp J-J, Favre L, Dupin JM, Spérandio M. A method for the quantitative evaluation of fish movements in salt ponds by acoustic telemetry. J Exp Mar Biol Ecol. 1990;141: 221–236. doi: 10.1016/0022-0981(90)90226-3

[19] Hasselman B. Package ‘nleqslv’;; 2018.

[20] O’Dor RK, Andrade Y, Webber DM, Sauer WHH, Roberts MJ, Smale MJ, et al. Applications and performance of radio-acoustic positioning and telemetry (RAPT) systems. In: Advances in invertebrates and fish telemetry. Dordrecht: Springer; 1998. pp. 1–8.

[21] Ransom BH, Steig TW, Timko MA, Nealson PA. Basin-wide monitoring of salmon smolts at US dams. Int J Hydropower Dams. 2008;15: 44–49.

[22] Nebiolo KP, Meyer TH. High precision 3-D coordinates for JSATS tagged fish in an acoustically noisy environment. Anim Biotelem. 2021;9: 1–15. doi: 10.1186/s40317-021-00244-0

[23] R Core Team. R: A Language and Environment for Statistical Computing; 2021. Available from: https://www.R-project.org/. Vienna, Austria: R Foundation for Statistical Computing.

[24] Takagi J, Ichikawa K, Arai N, Shoji J, Mitamura H. Challenge of monitoring cohesive movement in homing fish using fine-scale 3D positioning. Aquat Biol. 2021;30: 33–46. doi: 10.3354/ab00739

