# Supplementary material for: A simple intuitive method for seeking intersections of hyperbolas for acoustic positioning biotelemetry
Source: PLoS One. 2022 Nov 9;17(11):e0276289. doi: 10.1371/journal.pone.0276289 (PMC9645641; doi:10.1371/journal.pone.0276289)
Supplement: S1 File — (PDF) [file pone.0276289.s001.pdf]

# 1 Two-sheet hyperboroid of revolution

Two receivers A and B are deployed at $A(0,0,0)$ and $B(p,0,0)$, respectively. It is assumed that a transmitter emits a signal at a given point of $(x,y,z)$. Let $c(>0)$ be underwater sound speed. Let $t(\neq 0)$ be a time-difference-of-arrival (TDOA) between the receiver A and B. One obtains the following equation.

$$\sqrt{x^2+y^2+z^2} - \sqrt{(x-p)^2+y^2+z^2} = R \tag{1.1}$$

*where*

$$R = ct \neq 0 \qquad (\because c > 0, t \neq 0) \tag{1.2}$$

For (1.1), transposing the first term of the left-hand side to the right-hand side and squaring the two sides, one obtains

$$R^2 - b^2 - 2px = 2R\sqrt{x^2+y^2+z^2} \tag{1.3}$$

Further squaring the two sides, one obtains

$$Ax^2 - Apx + y^2 + z^2 = \frac{A^2R^2}{4} \tag{1.4}$$

*where*

$$A = 1 - \frac{p^2}{R^2} > 0 \qquad (\because p < R) \tag{1.5}$$

Dividing both side of (1.4) by $A$, and sorting out it, then one obtains

$$x^2 - px + \frac{y^2}{A} + \frac{z^2}{A} = \frac{AR^2}{4} \tag{1.6}$$

$$\Leftrightarrow (x - \frac{p}{2})^2 + \frac{y^2}{A} + \frac{z^2}{A} = \frac{1}{4}(AR^2 + p^2) \tag{1.7}$$

From (1.5), we obtain

$$AR^2 = R^2 - p^2 \tag{1.8}$$

Substituting (1.8) into (1.7), one obtains

$$\frac{(x-\frac{p}{2})^2}{R^2} - \frac{y^2}{p^2-R^2} - \frac{z^2}{p^2-R^2} = \frac{1}{4} \tag{1.9}$$
$$\Leftrightarrow \quad \frac{(x-\frac{p}{2})^2}{R^2} - \frac{y^2}{(\sqrt{p^2-R^2})^2} - \frac{z^2}{(\sqrt{p^2-R^2})^2} = \frac{1}{4} \tag{1.10}$$
$$\Leftrightarrow \quad \frac{(x-\frac{p}{2})^2}{(\frac{R}{2})^2} - \frac{y^2}{(\frac{\sqrt{p^2-R^2}}{2})^2} - \frac{z^2}{(\frac{\sqrt{p^2-R^2}}{2})^2} = 1 \tag{1.11}$$

Considering the equivalence of the coefficients of $y$ and $z$ and the sign of each variable, (1.11) represents a two-sheet hyperboloid of revolution generated by rotating a hyperbola around x-axis with foci of $(0, 0, 0)$ and $(p, 0, 0)$.

## 2 Coefficient for converting a hyperboloid to a hyperbola

With $a, b, c$ as parameters, the formula for a two-sheet hyperboloid of revolution is

$$\frac{x^2}{a^2} - \frac{y^2}{b^2} - \frac{z^2}{c^2} = 1 \tag{2.1}$$

From section one, it was known that one should consider a two-sheet hyperboloid of revolution generated by rotating a hyperbola around x-axis. The condition for it is that $c = b$ in (2.1). Let $z = z_0$ be the position in the z-axis where the transmitter is located, i.e. the plane defined by the difference between the depth of a transmitter and an installation depth of the receivers. Substituting $c = b$ and $z = z_0$ into (2.1), one obtains

$$\frac{x^2}{a^2} - \frac{y^2}{b^2} = 1 + \frac{{z_0}^2}{b^2} \tag{2.2}$$
$$\Leftrightarrow \quad \frac{x^2}{(a\sqrt{1+\frac{{z_0}^2}{b^2}})^2} - \frac{y^2}{(b\sqrt{1+\frac{{z_0}^2}{b^2}})^2} = 1 \tag{2.3}$$

This is an equation of a hyperbola obtained by slicing a hyperboloid in the plane $z = z0$. Therefore, a correction coefficient $\sigma$ at $z = z_0$ converting the hyperbolic parameters $a$ and $b$ is as follows.

$$\sigma = \sqrt{1 + \frac{{z_0}^2}{b^2}} > 1 \qquad (\because z_0 \geq 0, b > 0) \tag{2.4}$$

$\sigma$ gets greater as $z_0$ greater, so both parameters $a$ and $b$ also get greater. This means that, as $z_0$ gets greater, foci of a hyperbola leave from origin. The fact that the ratio of $a$ to $b$ remains unchanged represents that the asymptote also remains unchanged.
