# Supplementary material for: A simple intuitive method for seeking intersections of hyperbolas for acoustic positioning biotelemetry
Source: PLoS One. 2022 Nov 9;17(11):e0276289. doi: 10.1371/journal.pone.0276289 (PMC9645641; doi:10.1371/journal.pone.0276289)
Supplement: S2 File — (PDF) [file pone.0276289.s002.pdf]

The source codes of three positioning methods are shown below: (1) The proposed method, (2) An analytical method using a pencil, and (3) An approximating method using Newton-Raphson method. The codes were written in R language (R Core Team).

(1) Proposed Method

```
proposed_method <- function(Rx,Ry,Rz,c=1500,t,Tz=NA){
  # Rx,Ry,Rz: x,y,z coordinates of three receivers
  # c: underwater sound speed
  # t: record time in three receivers
  # Tz: z coordinate of transmitter

  TDOA <- t[2:3]-t[1]
  d <- sqrt((Rx[2:3]-Rx[1])^2+(Ry[2:3]-Ry[1])^2+(Rz[2:3]-Rz[1])^2)
  if (sum(abs(TDOA)>=d/c)>0){return(list(x=NA, y=NA)); break}

  a <- c*TDOA/2
  b <- sqrt((d/2)^2-a^2)
  if (!is.na(Tz)){
    z <- Rz[1]-Tz
    cor.coef <- sqrt(1+(z/b)^2)
    a <- a*cor.coef
    b <- b*cor.coef
  }
  delta.x <- (Rx[1]+Rx[2:3])/2
  delta.y <- (Ry[1]+Ry[2:3])/2
  phi <- atan2(Ry[1]-delta.y, Rx[1]-delta.x)

  # H2(x2,y2) on H2-coordinate system
  x2 <- a[2]/cos(theta)
  y2 <- b[2]*tan(theta)
  # H2(X2,Y2) on R-coordinate system
  X2 <- x2*cos(phi[2]) - y2*sin(phi[2]) + delta.x[2]
  Y2 <- x2*sin(phi[2]) + y2*cos(phi[2]) + delta.y[2]
  # H2'(x2',y2') on H1-coordinate system
  x2p <- (X2-delta.x[1])*cos(-phi[1]) - (Y2-delta.y[1])*sin(-phi[1])
  y2p <- (X2-delta.x[1])*sin(-phi[1]) + (Y2-delta.y[1])*cos(-phi[1])
  # H1(x1,y2') on H1-coordinate system
  x1 <- a[1]*sqrt(1 + y2p^2/b[1]^2)
  # Delta: x-axis distance on H1-coordinate system
  Delta <- abs(x1-x2p)
  # i: index of local minimum
  i <- which(diff(sign(diff(Delta)))==2)+1
  i <- i[which(Delta[i]<1)]
  if (length(i)>2){
    i <- i[-which.max(Delta[i])]
  }
  # Estimated position on R-coordinate system
  loc.x <- X2[i]
  loc.y <- Y2[i]

  return(list(x=loc.x, y=loc.y))
}
```

(2) Analytical Method

```
pencil_method <- function(Rx,Ry,Rz,c,t,Tz=NA){
  # Rx,Ry,Rz: x,y,z coordinates of three receivers
  # c: underwater sound speed
  # t: record time in three receivers
  # Tz: z coordinate of transmitter

  R_ab <- c*(t[1]-t[2])
  R_ac <- c*(t[1]-t[3])
  if (is.na(Tz)){z <- 0}else{z <- Rz[1]-Tz}

  tmp <- Rx^2+Ry^2
  A <- tmp[1]
  B <- tmp[2]
  C <- tmp[3]
  D <- (R_ab*(Rx[3]-Rx[1])-R_ac*(Rx[2]-Rx[1])) / (R_ac*(Ry[2]-Ry[1])-R_ab*(Ry[3]-Ry[1]))
  E <- (R_ab*(R_ac^2+A-C)-R_ac*(R_ab^2+A-B)) / (2*(R_ac*(Ry[2]-Ry[1])-R_ab*(Ry[3]-Ry[1])))
  P <- (Rx[2]-Rx[1]) + D*(Ry[2]-Ry[1])
  Q <- 2*E*(Ry[2]-Ry[1]) + R_ab^2 + A - B
  F <- (P / R_ab)^2 -D^2 -1
  G <- ((P*Q) / R_ab^2) - 2*(D*(E - Ry[1]) - Rx[1])
  H <- (Q / (2*R_ab))^2 - A - E^2 + 2*Ry[1]*E

  x <- (-G + c(sqrt(G^2 - 4*F*(H-z^2)), -sqrt(G^2 - 4*F*(H-z^2)))) / (2*F)
  y <- D*x + E

  sign <- (sqrt((x-Rx[1])^2+(y-Ry[1])^2+z^2) - sqrt((x-Rx[2])^2+(y-Ry[2])^2+z^2)) * R_ab
  i <- which(sign>0)
  return(list(x=x[i], y=y[i], slope=D, intercept=E))
}
```

(3) Approximating Method

```
Newton_method <- function(Rx,Ry,Rz,c,t,Tz=NA){
  # Rx,Ry,Rz: x,y,z coordinates of three receivers
  # c: underwater sound speed
  # t: record time in three receivers
  # Tz: z coordinate of transmitter

  R <- c*(t[2:3]-t[1])
  if (is.na(Tz)){z <- 0}else{z <- Rz[1]-Tz}

  f <- function(x,y){
         sqrt((x-Rx[2])^2+(y-Ry[2])^2+z^2) - sqrt((x-Rx[1])^2+(y-Ry[1])^2+z^2) - R[1]
      }
  g <- function(x,y){
         sqrt((x-Rx[3])^2+(y-Ry[3])^2+z^2) - sqrt((x-Rx[1])^2+(y-Ry[1])^2+z^2) - R[2]
      }

  fx <- function(x,y){
         (x-Rx[2])/sqrt((x-Rx[2])^2+(y-Ry[2])^2+z^2) –
         (x-Rx[1])/sqrt((x-Rx[1])^2+(y-Ry[1])^2+z^2)
       }
  fy <- function(x,y){
         (y-Ry[2])/sqrt((x-Rx[2])^2+(y-Ry[2])^2+z^2) –
         (y-Ry[1])/sqrt((x-Rx[1])^2+(y-Ry[1])^2+z^2)
       }
  gx <- function(x,y){
         (x-Rx[3])/sqrt((x-Rx[3])^2+(y-Ry[3])^2+z^2) –
         (x-Rx[1])/sqrt((x-Rx[1])^2+(y-Ry[1])^2+z^2)
       }
  gy <- function(x,y){
         (y-Ry[3])/sqrt((x-Rx[3])^2+(y-Ry[3])^2+z^2) –
         (y-Ry[1])/sqrt((x-Rx[1])^2+(y-Ry[1])^2+z^2)
       }

  x <- mean(Rx)
  y <- mean(Ry)
  j <- 0
  idx <- c(1,1)
  e <- 0.001
  nlim <- 5
  while (sum(idx<e)!=2 & j<nlim) {
    A <- matrix(c(fx(x,y), gx(x,y), fy(x,y), gy(x,y)),2,2)
    B <- matrix(c(-f(x,y), -g(x,y)),2,1)
    delta <- solve(a=A, b=B)
    idx <- c(abs(delta[1]/x), abs(delta[2]/y))
    x <- x + delta[1]
    y <- y + delta[2]
    j <- j+1
  }

  return(list(x=x, y=y))
}
```
