# Supplementary material for: A simple intuitive method for seeking intersections of hyperbolas for acoustic positioning biotelemetry
Source: PLoS One. 2022 Nov 9;17(11):e0276289. doi: 10.1371/journal.pone.0276289 (PMC9645641; doi:10.1371/journal.pone.0276289)
Supplement: S4 File — (PDF) [file pone.0276289.s004.pdf]

1 3-D positioning using four receivers without depth information of a transmitter

We shall describe a positioning method in 3-D space. Assuming that four receivers A(a_x, a_y, a_z), B(b_x, b_y, b_z), C(c_x, c_y, c_z), and D(d_x, d_y, d_z) are deployed in 3-D space. Let T_a, T_b, T_c, and T_d be arrival time of a signal from a transmitter, respectively. Thus time-difference-of-arrivals (TDOA) between A and B, A and C, and A and D are $T_{ab} = T_a - T_b \neq 0$, $T_{ac} = T_a - T_c \neq 0$, and $T_{ad} = T_a - T_d \neq 0$, respectively. Let c be underwater sound speed. The coordinate (x, y, z) of the transmitter position suffices the following equations.

$$\sqrt{(x-a_x)^2+(y-a_y)^2+(z-a_z)^2} - \sqrt{(x-b_x)^2+(y-b_y)^2+(z-b_z)^2} = R_{ab} \tag{1.1}$$

$$\sqrt{(x-a_x)^2+(y-a_y)^2+(z-a_z)^2} - \sqrt{(x-c_x)^2+(y-c_y)^2+(z-c_z)^2} = R_{ac} \tag{1.2}$$

$$\sqrt{(x-a_x)^2+(y-a_y)^2+(z-a_z)^2} - \sqrt{(x-d_x)^2+(y-d_y)^2+(z-d_z)^2} = R_{ad} \tag{1.3}$$

where

$$R_{ab} = cT_{ab}$$
$$R_{ac} = cT_{ac}$$
$$R_{ad} = cT_{ad}$$

Each equation represents one sheet of two-sheets hyperboloid. In each equation, transposing the first term of the left-hand side to the right-hand side, and squaring the two side, one obtains

$$\frac{1}{2}({R_{ab}}^2 + A - B) + (b_x - a_x)x + (b_y - a_y)y + (b_z - a_z)z - R_{ab}\sqrt{(x-a_x)^2+(y-a_y)^2+(z-a_z)^2} = 0 \tag{1.4}$$

$$\frac{1}{2}({R_{ac}}^2 + A - C) + (c_x - a_x)x + (c_y - a_y)y + (c_z - a_z)z - R_{ac}\sqrt{(x-a_x)^2+(y-a_y)^2+(z-a_z)^2} = 0 \tag{1.5}$$

$$\frac{1}{2}({R_{ad}}^2 + A - D) + (d_x - a_x)x + (d_y - a_y)y + (d_z - a_z)z - R_{ad}\sqrt{(x-a_x)^2+(y-a_y)^2+(z-a_z)^2} = 0 \tag{1.6}$$

where

$$A = {a_x}^2 + {a_y}^2 + {a_z}^2$$
$$B = {b_x}^2 + {b_y}^2 + {b_z}^2$$
$$C = {c_x}^2 + {c_y}^2 + {c_z}^2$$
$$D = {d_x}^2 + {d_y}^2 + {d_z}^2$$

Here, apply a concept of a pencil to equations. Let (1.4), (1.5), and (1.6) be $f(x, y, z) = 0, g(x, y, z) = 0, h(x, y, z) = 0$, respectively. One obtains

$$f(x,y,z) - \frac{R_{ab}}{R_{ac}} g(x,y,z) = 0 \tag{1.7}$$

$$f(x,y,z) - \frac{R_{ab}}{R_{ad}} h(x,y,z) = 0 \tag{1.8}$$

Substituting (1.4), (1.5), and (1.6) to (1.7) and (1.8), and arranging each equation, one obtains

$$\Big(R_{ac}(b_x - a_x) - R_{ab}(c_x - a_x)\Big)x + \Big(R_{ac}(b_y - a_y) - R_{ab}(c_y - a_y)\Big)y + \Big(R_{ac}(b_z - a_z) - R_{ab}(c_z - a_z)\Big)z + \frac{1}{2}\Big(R_{ac}({R_{ab}}^2 + A - B) - R_{ab}({R_{ac}}^2 + A - C)\Big) = 0 \tag{1.9}$$

$$\Big(R_{ad}(b_x - a_x) - R_{ab}(d_x - a_x)\Big)x + \Big(R_{ad}(b_y - a_y) - R_{ab}(d_y - a_y)\Big)y + \Big(R_{ad}(b_z - a_z) - R_{ab}(d_z - a_z)\Big)z + \frac{1}{2}\Big(R_{ad}({R_{ab}}^2 + A - B) - R_{ab}({R_{ad}}^2 + A - D)\Big) = 0 \tag{1.10}$$

Equation (1.9) and (1.10) are linear expressions in x, y, z, so they are planes in 3-D space. Arranging each equation, one obtains

$$y = Ex + Fz + G \tag{1.11}$$

$$y = Hx + Iz + J \tag{1.12}$$

$$where$$

$$E = \frac{R_{ab}(c_x - a_x) - R_{ac}(b_x - a_x)}{R_{ac}(b_y - a_y) - R_{ab}(c_y - a_y)}$$

$$F = \frac{R_{ab}(c_z - a_z) - R_{ac}(b_z - a_z)}{R_{ac}(b_y - a_y) - R_{ab}(c_y - a_y)}$$

$$G = \frac{R_{ab}({R_{ac}}^2 + A - C) - R_{ac}({R_{ab}}^2 + A - B)}{2\Big(R_{ac}(b_y - a_y) - R_{ab}(c_y - a_y)\Big)}$$

$$H = \frac{R_{ab}(d_x - a_x) - R_{ad}(b_x - a_x)}{R_{ad}(b_y - a_y) - R_{ab}(d_y - a_y)}$$

$$I = \frac{R_{ab}(d_z - a_z) - R_{ad}(b_z - a_z)}{R_{ad}(b_y - a_y) - R_{ab}(d_y - a_y)}$$

$$J = \frac{R_{ab}({R_{ad}}^2 + A - D) - R_{ad}({R_{ab}}^2 + A - B)}{2\Big(R_{ad}(b_y - a_y) - R_{ab}(d_y - a_y)\Big)}$$

Find the line where these planes intersect. From (1.11) and (1.12),

$$
\begin{aligned}
&Ex + Fz + G = Hx + Iz + J && (1.13)\\
&\Leftrightarrow x = Kz + L && (1.14)\\
&where\\
&K = \frac{I - F}{E - H}\\
&L = \frac{J - G}{E - H}
\end{aligned}
$$

Substituting (1.14) into (1.11), one obtains

$$
\begin{aligned}
&y = E(Kz + L) + Fz + G && (1.15)\\
&\Leftrightarrow y = Mz + N && (1.16)\\
&where\\
&M = EK + F\\
&N = EL + G
\end{aligned}
$$

Based on the above, coordinate of the line where the two plane intersect is $(Kz+L, Mz+N, z)$. Find the intersection between this line and hyperboloid $f(x, y, z) = 0$, i.e., (1.4). Substituting (1.14) and (1.16) into (1.4), one obtains

$$
\begin{aligned}
&\frac{1}{2}({R_{ab}}^2 + A - B) + (b_x - a_x)(Kz + L) + (b_y - a_y)(Mz + N) + (b_z - a_z)z\\
&-R_{ab}\sqrt{((Kz + L) - a_x)^2 + ((Mz + N) - a_y)^2 + (z - a_z)^2} = 0 && (1.17)\\
&\Leftrightarrow Oz + P = R_{ab}\sqrt{(K^2 + M^2 + 1)z^2 + 2(KL + MN - a_xK - a_yM - a_z)z + A - 2(a_xL - a_yN)} && (1.18)\\
&where\\
&O = (b_x - a_x)K + (b_y - a_y)M + (b_z - a_z)\\
&P = \frac{1}{2}({R_{ab}}^2 + A - B) + (b_x - a_x)L + (b_y - a_y)N
\end{aligned}
$$

Squaring both side of (1.18) and arranging it, one obtains

$$
\begin{aligned}
&O^2z^2 + 2OPz + P^2 = {R_{ab}}^2((K^2 + M^2 + 1)z^2 + 2(KL + MN - a_xK - a_yM - a_z)z + A - 2(a_xL - a_yN)) && (1.19)\\
&\Leftrightarrow Qz^2 + Sz + T = 0 && (1.20)\\
&where\\
&Q = {R_{ab}}^2(K^2 + M^2 + 1) - O^2\\
&S = 2({R_{ab}}^2(KL + MN - a_xK - a_yM - a_z) - OP)\\
&T = {R_{ab}}^2(A + L^2 + N^2 - 2(a_xL + a_yN)) - P^2
\end{aligned}
$$

Applying the quadratic formula to (1.20) to find z, one obtains

$$z = \frac{-S \pm \sqrt{S^2 - 4QT}}{2Q} \tag{1.21}$$

Finding x and y by substituting (1.21) respectively into (1.14) and (1.16), one obtains two candidate for the intersection (Fig. 1). In the case that all the installation depth (z coordinate) of four receivers is not the same, intersection(s) can be selected by considering a condition. For example, substituting (x, y, z) into (1.4), the pair holding the equality is the coordinate of the intersection(s). In the case that there is only one intersection, only one pair of (x, y, z) holds the equality. The other pair represents an intersection of the line and the other sheet of the hyperboloid $f(x, y, z) = 0$. In the case that there are two intersections, both two pair of (x, y, z) hold the equality. In the case that all the installation depth (z coordinate) of four receivers is the same, no intersection can be selected because the solutions have z being symmetrical against the xy-plane.

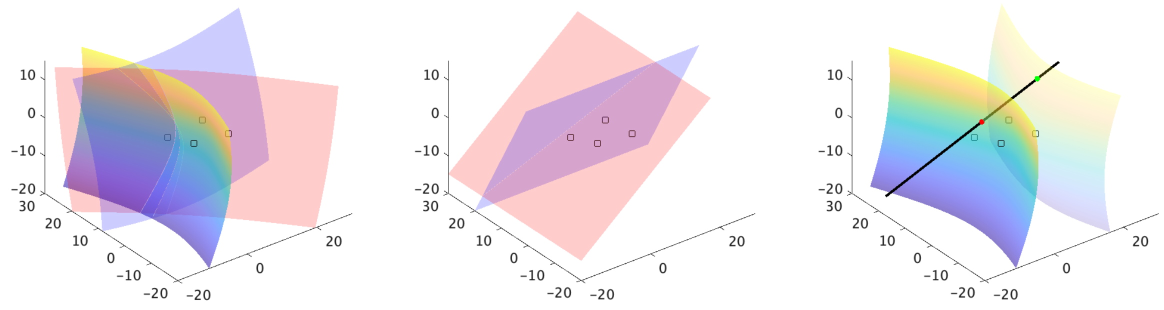

図 1 3-D image of positioning method using a pencil. Two sheets (Center) can be made from two combination of three hyperboloid (Left). The black line (Right) represents an intersecting line between the two sheet. Intersection of the three hyperboloid is at the red point where the line crosses one sheet of the hyperboloid (Righit). The other intersection is at the green point where the line crosses the other sheet of the hyperboloid (Righit).

2 3-D positioning using three receivers of which installation depth is not the same with depth information of a transmitter

We shall describe another positioning method in 3-D space. Assuming that three receivers $A(a_x, a_y, a_z)$, $B(b_x, b_y, b_z)$, and $C(c_x, c_y, c_z)$ are deployed in 3-D space. Let T_a, T_b, and T_c be arrival time of a signal from a transmitter, respectively. Thus time-difference-of-arrivals (TDOA) between A and B, and A and C are $T_{ab} = T_a - T_b \neq 0$, and $T_{ac} = T_a - T_c \neq 0$, respectively. Let c be underwater sound speed. The

coordinate (x, y, z) of the transmitter position suffices the following equations.

$$\sqrt{(x-a_x)^2+(y-a_y)^2+(z-a_z)^2}-\sqrt{(x-b_x)^2+(y-b_y)^2+(z-b_z)^2}=R_{ab} \tag{2.1}$$

$$\sqrt{(x-a_x)^2+(y-a_y)^2+(z-a_z)^2}-\sqrt{(x-c_x)^2+(y-c_y)^2+(z-c_z)^2}=R_{ac} \tag{2.2}$$

where

$$R_{ab} = cT_{ab}$$
$$R_{ac} = cT_{ac}$$

Each equation represents one blanch of a hyperbola, i.e., a hyperbolic LOP. In each equation, transposing the first term of the left-hand side to the right-hand side, and squaring the two side, one obtains

$$\frac{1}{2}({R_{ab}}^2+A-B)+(b_x-a_x)x+(b_y-a_y)y+(b_z-a_z)z-R_{ab}\sqrt{(x-a_x)^2+(y-a_y)^2+(z-a_z)^2}=0 \tag{2.3}$$

$$\frac{1}{2}({R_{ac}}^2+A-C)+(c_x-a_x)x+(c_y-a_y)y+(c_z-a_z)z-R_{ac}\sqrt{(x-a_x)^2+(y-a_y)^2+(z-a_z)^2}=0 \tag{2.4}$$

where

$$A = {a_x}^2+{a_y}^2+{a_z}^2$$
$$B = {b_x}^2+{b_y}^2+{b_z}^2$$
$$C = {c_x}^2+{c_y}^2+{c_z}^2$$

Here, apply a concept of a pencil to equations. Let (2.1), and (2.2) be $f(x, y, z) = 0$, and $g(x, y, z) = 0$, respectively. One obtains

$$f(x,y,z)-\frac{R_{ab}}{R_{ac}}g(x,y,z)=0 \tag{2.5}$$

Equation (2.5) is linear expressions in x, y, z, so it is a plane in 3-D space. Arranging this equation, one obtains

$$y = Dx + Ez + F \tag{2.6}$$

where

$$D = \frac{R_{ab}(c_x - a_x) - R_{ac}(b_x - a_x)}{R_{ac}(b_y - a_y) - R_{ab}(c_y - a_y)}$$

$$E = \frac{R_{ab}(c_z - a_z) - R_{ac}(b_z - a_z)}{R_{ac}(b_y - a_y) - R_{ab}(c_y - a_y)}$$

$$F = \frac{R_{ab}({R_{ac}}^2 + A - C) - R_{ac}({R_{ab}}^2 + A - B)}{2\Big(R_{ac}(b_y - a_y) - R_{ab}(c_y - a_y)\Big)}$$

From the above, coordinate of the line where this plane and the plane with the transmitter $z = z_0$ intersect is $(Kz + L, Mz + N, z)$. Substituting this coordinate into (2.3), one obtains

$$\frac{1}{2}({R_{ab}}^2 + A - B) + (b_x - a_x)x + (b_y - a_y)(Dx + Ez_0 + F) + (b_z - a_z)z_0$$

$$-R_{ab}\sqrt{(x - a_x)^2 + ((Dx + Ez_0 + F) - a_y)^2 + (z_0 - a_z)^2} = 0 \tag{2.7}$$

$$\Leftrightarrow Gx + H = R_{ab}\sqrt{(1 + D^2)x^2 + 2((Ez_0 + F - a_y)D - a_x)x + {a_x}^2 + (Ez_0 - F - a_y)^2 + (z_0 - a_z)^2} \tag{2.8}$$

where

$$G = (b_x - a_x) + (b_y - a_y)D$$

$$H = \frac{1}{2}({R_{ab}}^2 + A - B) + ((b_z - a_z) + (b_y - a_y)E)z_0 + (b_y - a_y)F$$

Squaring both two sides of (2.8) and arranging it, one obtains

$$G^2x^2 + 2GHx + H^2 = {R_{ab}}^2((1 + D^2)x^2 + 2((Ez_0 + F - a_y)D - a_x)x + {a_x}^2 + (Ez_0 - F - a_y)^2 + (z_0 - a_z)^2) \tag{2.9}$$

$$\Leftrightarrow Ix^2 + Jx + K = 0 \tag{2.10}$$

where

$$I = {R_{ab}}^2(1 + D^2) - G^2$$

$$J = 2({R_{ab}}^2((Ez_0 + F - a_y)D - a_x) - GH)$$

$$K = {R_{ab}}^2({a_x}^2 + (Ez_0 + F - a_y)^2 + (z_0 - a_z)^2 - 2z_0a_z)) - H^2$$

Applying the quadratic formula to (2.10) to find x, one obtains

$$x = \frac{-J \pm \sqrt{J^2 - 4IK}}{2I} \tag{2.11}$$

Substituting x and z_0 into (2.6) to find y, one obtains two candidate for the intersection (Fig. 2).

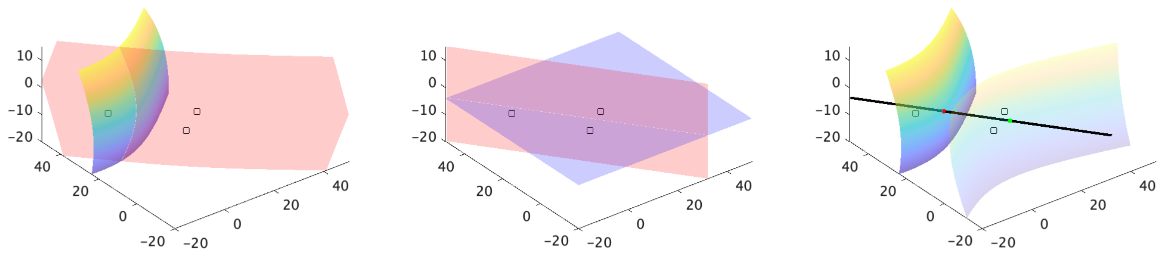

図 2 3-D image of another positioning method using a pencil. One red sheet (Center) can be made from two hyperboloid (Left). The black line (Right) represents an intersecting line between the red sheet and the depth plane of the blue sheet (Center). Intersection of the two hyperboloid is at the red point where the line crosses one sheet of the hyperboloid (Righit). The other intersection is at the green point where the line crosses the other sheet of the hyperboloid (Righit).
